# The Spatial Distribution of *Mustelidae* in France

**DOI:** 10.1371/journal.pone.0121689

**Published:** 2015-03-26

**Authors:** Clément Calenge, Joël Chadoeuf, Christophe Giraud, Sylvie Huet, Romain Julliard, Pascal Monestiez, Jérémy Piffady, David Pinaud, Sandrine Ruette

**Affiliations:** 1 Office national de la chasse et de la faune sauvage, Direction des études et de la recherche, Saint Benoist, BP 20. 78612 Le Perray en Yvelines, France; 2 Statistics, UR1052, Domaine Saint Maurice 67, Allée des chênes CS 60094 F-84143 Montfavet cedex, France; 3 CMAP, UMR 7641, Ecole Polytechnique, Palaiseau, France—Laboratoire de Mathématiques d’Orsay, UMR 8628, Université Paris-Sud, Orsay, France; 4 UR 341 MIA, INRA, F78352, Jouy-en-Josas, France; 5 CESCO, UMR 7204, MNHN-CNRS-UPMC, CP51, 55 rue Buffon, 75005 Paris, France; 6 INRA—Unité BioSp. Domaine Saint-Paul, Site Agroparc, 84914 Avignon Cedex 9, France; 7 IRSTEA, UR MALY, centre de Lyon-Villeurbanne F-69626 Villeurbanne, France; 8 CEBC, UMR 7372—CNRS/Univ La Rochelle, 79360 Villiers en Bois, France; Institut Pluridisciplinaire Hubert Curien, FRANCE

## Abstract

We estimated the spatial distribution of 6 *Mustelidae* species in France using the data collected by the French national hunting and wildlife agency under the “small carnivorous species logbooks” program. The 1500 national wildlife protection officers working for this agency spend 80% of their working time traveling in the spatial area in which they have authority. During their travels, they occasionally detect dead or living small and medium size carnivorous animals. Between 2002 and 2005, each car operated by this agency was equipped with a logbook in which officers recorded information about the detected animals (species, location, dead or alive, date). Thus, more than 30000 dead or living animals were detected during the study period. Because a large number of detected animals in a region could have been the result of a high sampling pressure there, we modeled the number of detected animals as a function of the sampling effort to allow for unbiased estimation of the species density. For dead animals -- mostly roadkill -- we supposed that the effort in a given region was proportional to the distance traveled by the officers. For living animals, we had no way to measure the sampling effort. We demonstrated that it was possible to use the whole dataset (dead and living animals) to estimate the following: (i) the relative density -- i.e., the density multiplied by an unknown constant -- of each species of interest across the different French agricultural regions, (ii) the sampling effort for living animals for each region, and (iii) the relative detection probability for various species of interest.

## Introduction

All *Mustelidae* species endemic to France are listed as strictly protected species in Appendix II (*Mustela lutreola*, *Lutra lutra*) or as protected species in Appendix III of the Bern convention on the conservation of European Wildlife and Natural Habitats. However, some of these species are still regarded as pests in some regions of the signing countries, either because they can be carriers of dangerous diseases for human and cattle or because they may cause important damage to human activities. For these reasons, the Bern convention does not forbid hunting and other types of exploitation of these protected species, as long as their populations are at “a level which corresponds in particular to ecological, scientific and cultural requirements” (article 2 of the convention), in order “to keep the populations out of danger” (article 7).

Whether the density of a given species reaches the “required population level” in a given place is typically determined by the wildlife manager. He generally knows the conservation situation of the species in a few places fairly well, and he uses those places as “references” to which other places are compared to evaluate the situation of the species in the whole area of interest (while considering other measures of the species situation as well, e.g. damage to livestock, the number of trapped animals). Because the density estimates are commonly used for a comparison between various places, it is ordinarily not necessary to work with “absolute” density measurements. In many cases, working with an index proportional to the actual density of the species—which we call “relative density”—is sufficient for sensible wildlife management [1, 2].

In addition, relative density is much easier to estimate than absolute density. On the one hand, most statistical methods developed for the estimation of the absolute density rely on cumbersome designs—e.g., mark-recapture methods, distance sampling approaches, or removal models [3, 4]. Such approaches are far too expensive to be considered for the estimation of the density of all *Mustelidae* species in every region at the scale of a whole country. On the other hand, the relative density can be estimated from much simpler designs: a set of sites is randomly sampled in the area of interest, and counts of organisms are organized for these sites. At a given site, the resulting count can be used as a proxy of the true density (see [5] for an example). Indeed, if the assumption of constant detectability holds over space and time, the average number of animals counted per sampled site is proportional to the true density of the species in the area [1]. Thus, estimates of relative density have been commonly used to map the spatial distribution of several species [6].

However, such sampling designs developed for the estimation of the relative density are difficult to implement for *Mustelidae* species in France: those species are shy, and most of them are nocturnal and therefore difficult to detect. The stoat *Mustela ermina* and the weasel *Mustela nivalis* are partly diurnal, but these two species are hardly detectable because they are much smaller than the other *Mustelidae*. Finally, as for many carnivorous species, their densities are low in comparison to the species generally monitored with such designs (e.g., common birds). The monitoring of carnivorous species is thus a complex task.

For this reason, most authors in the literature have recommended to monitor carnivorous species with the help of so-called “field signs”, such as track counts [7] or faecal counts [8, 9]. However, the use of field signs is generally associated with serious issues with species identification. For example, it is very difficult to determine with certainty whether a footprint belongs to a stoat or a weasel [10]; in studies relying on faecal counts, even highly trained experts may mis-identify a large proportion of faeces [11]. Moreover, the use of a given type of field sign can be sensible for a species, but not for another (see [10]). Finally, index scores based on field signs are likely to be more reliable when used to approximate population trends over time in one location than when used to compare between areas [12]. Yet field signs are usually preferred over other approaches relying on direct or indirect counts of animals, such as spotlight counts [13] or trapping statistics [14, 15]. Such counts are considered as impractical because they are too costly and not sensitive enough for a large-scale monitoring [16, 17].

However, we show in this paper that direct and indirect counts can also be collected at low cost to derive meaningful estimates of the relative density, thereby avoiding—to some extent (see discussion) – most of the issues raised by the use of field signs. Thus, in 2002–2005, the French national hunting and wildlife agency (Office national de la chasse et de la faune sauvage, hereafter ONCFS) implemented a plan to collect occurrence data on *Mustelidae* species. The “small carnivorous species logbooks” program (hereafter SCSL program) relied on the important workforce of this organization. Indeed, the 1500 national wildlife protection officers working for this organization spend a lot of time in the field and therefore occasionally detect living or dead *Mustelidae*. During the study period, each official car was equipped with a logbook in which information concerning each animal detected by the wildlife protection officers was recorded (i.e., the species, the municipality, the date of detection, and whether the animal was dead or alive).

The collected data could not be directly used to estimate relative density indices for the species of interest in the different French regions. Indeed, the number of detected animals in a given region partly depended on the time spent by the officers in this region. A large number of detected animals in a given region might have been the result of a high abundance of the species as well as the result of a strong observation effort (also called sampling effort in this paper) there. A model of the observation process was necessary to account for this source of variation in the abundance estimation [18]. Moreover, the observation process was not the same for dead and living animals. Living animals were often detected during the officers’ daytime operations in the field, whereas dead animals were often detected alongside the road (due to roadkill) during officers movements. Finally, for a given status (dead or alive), the detection probably varied among species because of their size variation.

**Fig 1:**
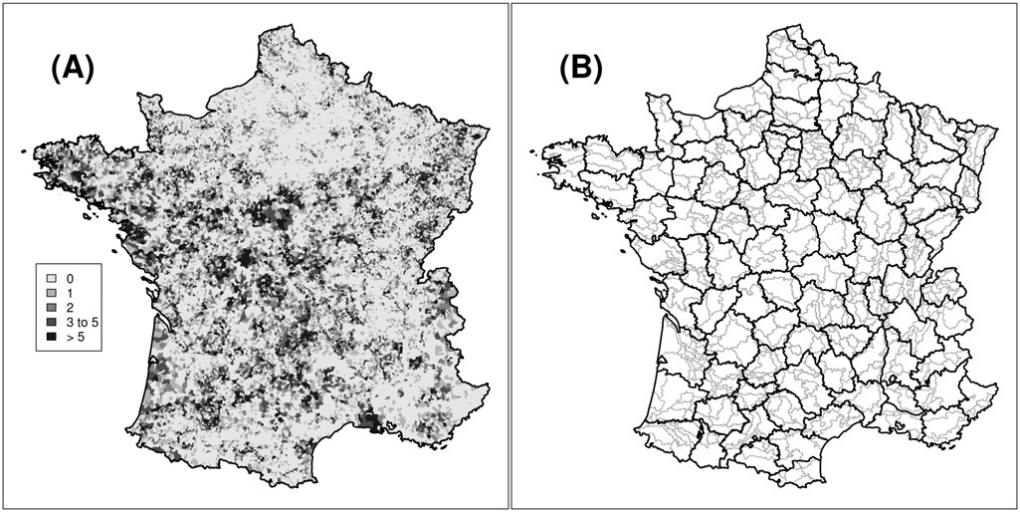
Data used for the estimation of the relative density of small carnivorous species in France. (A) number of detected animals in each municipality of France from 2002 to 2005 (the inset contains the legend). (B) Map of the small agricultural regions (SAR, in grey), and of the department (black)

In this paper, we used the data collected under the SCSL program to estimate the average animal density in spatial units characterized by homogeneous agricultural practices and activities (small agricultural regions) in France (Fig. 1B). Based on a model of the sampling effort for the dead animals, it was possible to estimate the relative density of each species in each region of interest using a log-linear model. We also took into account the expected spatial regularity of species density, as well as the effect of the environment on these densities, using a ridge-type regularization. We provide an R package named scsl (see S1 Package). This package contains the data, the C code and the R functions used for the calculations carried out in this paper. We also provide a document written using the Knitr system [19], describing the model fit and evaluation with these functions, as well as additional analyses carried out to demonstrate the robustness of our findings.

## Methods

### Data collection

The wildlife protection officers, who are in charge of both the wildlife protection and the hunting police, are officially designated agents commissioned by the French Minister of Ecology. Most of them are assigned to a department (a French administrative unit, see Fig. 1B). These officers spend about 80% of their working time in the field. They carry out two broad types of activity: (i) “static activities” during which officers spend a lot of time out of their car in a given municipality (e.g., wildlife monitoring, data collection for research, stakeout to arrest poachers) and (ii) “moving activities” during which officers spend a lot of time driving their car (e.g. police patrol missions). In general, a given team of officers (with generally two officers per team) always drives the same car. From 2001 to 2005, the SCSL program took advantage of this time spent by the officers in static and moving activities.

Our study period covers all years from 2002 to 2005. We did not take into account the first year of monitoring (2001) because the SCSL program was not yet well established at this time. We supposed that the species densities were constant during the study period (a reasonable hypothesis given the short time period, but see the discussion). During our study period, every car of the ONCFS was equipped with a logbook collected every year. The 1500 wildlife protection officers of this organization recorded therein each detected animal belonging to the following carnivorous species: (i) the stoat *Mustela erminea*, (ii) the weasel *Mustela nivalis*, (iii) the polecat *Mustela putorius*, (iv) the badger *Meles meles*, (v) the pine marten *Martes martes*, (vi) the stone marten *Martes foina*, (vii) the common genet *Genetta genetta* and (viii) the wildcat *Felis silvestris*. Note that the last two species are not *Mustelidae* and were not of direct interest in our study. For each detected animal, the officers noted down in the logbook the species, the status of the animal (dead or alive), the date of the detection and the municipality where the detection occurred. When a dead animal was detected several times, only one sighting was reported. Thus, during our study period, 31 811 animals belonging to these 8 species were recorded (Fig. 1A).

Considering the detection process, two broad groups of species can be distinguished. On the one hand, because of their small size, stoats and weasels were rarely seen dead, as their bodies rapidly degrade in the environment or disappear due to scavenging. Moreover, because they are partly diurnal, they could be detected alive during the daytime (more than 75% of stoats and weasels were indeed detected alive). According to an informal poll conducted among wildlife conservation officers, living stoats and weasels were detected mostly during static activities, and very rarely from a moving car. On the other hand, the other species (badgers, martens, etc.) are larger, and their bodies take a longer time to be degraded. Moreover, because all these species are nocturnal, they could be detected alive only during the agents’ nighttime missions. Actually, these species were most often roadkill detected during moving activities (74% of these animals were indeed detected dead). We assumed that there were no identification errors (see discussion on this issue).

For 51% of the car-years *c*, the distance *k*_*c*_ traveled by the car during the year was also reported in the logbook at the end of the year. We supposed that the probability of reporting the distance traveled by a given car did not depend on *k*_*c*_ (e.g., highly used cars were not more likely to report their traveled distance than less used cars). Under this assumption, and given that all the cars restrained their movements within the boundaries of the department in which the officer team worked, it was possible to roughly estimate the total number of kilometers *T*_*d*_ traveled by all cars during the study period in a given department *d*:

$$\begin{array}{c} T_{d}=B_{d}\times \frac{1}{R_{d}}\sum_{c=1}^{R_{d}}k_{c} \end{array}$$ (1)

where *B*_*d*_ is the total number of logbooks available in the department *d* during the study period and *R*_*d*_ is the number of logbooks available in this department for which the yearly distance traveled was also recorded. Note that the measure *T*_*d*_ implicitly accounts for the between-year variation of the number of kilometers traveled by a given car, as these numbers of kilometers are summed over the years.

### Basic modelling framework

The spatial unit of interest in our study was the “small agricultural region”. The SARs are defined by the intersection between the “large agricultural region” and the department. Each large agricultural region corresponds to a group of neighboring municipalities, possibly belonging to different departments, characterized by a homogeneous agricultural practices and activities (the 429 large agricultural regions are defined by the French ministry of agriculture; for further details see http://www.agreste.agriculture.gouv.fr/definitions/zonages/). The intersection between the 90 departments and the 429 large agricultural regions defines 703 small agricultural regions (SAR, Fig. 1B). Each department contains 7.8 SARs in average (SD = 3.3). The SARs are sufficiently small and homogeneous to be used for wildlife management purposes, but large enough to ensure that most of them (95%) contained at least one detected animal. Our aim was therefore to use the data collected under the SCSL program to estimate the density of the 6 *Mustelidae* species of interest in the 703 SARs of France.

Let *N*_*ijk*_ be the total number of animals of the species *i* (*i* = 1…*I*) in the SAR *j* (*j* = 1…*J*) with status *k* (*k* = 1 corresponding to dead animals and *k* = 2 corresponding to living animals) detected during the complete study period (i.e., the sum, over the years of the study period, of the annual counts for this particular combination *i*,*j*,*k*). First of all, we supposed that *N*_*ijk*_ could be described using a Poisson distribution:

$$\begin{array}{c} N_{ijk}∼𝒫\left(\lambda_{ijk}\right) \end{array}$$

where *λ*_*ijk*_ is the expectation of this distribution. Moreover, we supposed the following model for this expectation:

$$\begin{array}{c} \lambda_{ijk}=S_{j}A_{ij}E_{jk}P_{ik} \end{array}$$ (2)

where *S*_*j*_ is the known surface area of the *j* ^th^ SAR, *A*_*ij*_ is the true density of the species *i* in the SAR *j*, *E*_*jk*_ is the sampling effort in the SAR *j* for animals in the status *k*, and *P*_*ik*_ is the detection probability of the species *i* in the status *k*. Note that we used the terms “observational effort” and “sampling effort” interchangeably in this paper. Thus, the main assumption underlying this model is that, for a given status (dead or living animals), the observational process can be decomposed in two statistically independent components: an observational effort depending on the site only, and a detection probability depending only on the species.

The terms “sampling effort” and “detection probability” are just conventions here; the point is that, for a given status, no component of the observation process varied across both the sites and the species. For example, the detectability of a living animal was probably systematically lower in a SAR with a dense coverage of herbaceous cover than in a SAR characterized by a high coverage of bare ground. However, the component *E*_*jk*_ would accounts for such detectability differences between the sites. On the other hand, this assumption would be violated if some species were more easily detectable than others in some sites, but less detectable in other sites. This could occur, for example, if attention for some species was higher in some areas than in others. However, given the high homogeneity of the network in terms of duties and training, and the simplicity of the protocol (every observation should have been recorded), we considered it to be unlikely.

The model (2) belongs to the family of generalized linear models with multiplicative interactions [20]. As we formulated the problem here, the model (2) is degenerated in the sense that all the unknown parameters *A*_*ij*_,*E*_*jk*_ and *P*_*ik*_ are not uniquely identifiable. An infinity of parameter combinations could yield the same value of *λ*_*ijk*_. However, some parameters related to the sampling effort are actually known. Indeed, because dead animals were essentially roadkill detected during moving activities, the sampling effort of dead animals in a given SAR could be considered to be proportional to the total number of kilometers traveled by the officers in this SAR. As we will see later, it is possible to estimate this number of kilometers from the information available in the logbooks. On the other hand, we had no information on the sampling effort of living animals, which was proportional to the time spent by the officers on static activities—a typically unknown quantity.

We supposed thereafter that the number of kilometers *V*_*j*_ traveled by the officers in the *j* ^th^ SAR was known and proportional to the actual sampling effort *E*_*j*1_ for dead animals, i.e.,

$$\begin{array}{c} E_{j1}=\beta V_{j} \end{array}$$

with *β* an unknown constant. We reparametrized the model:

$$\begin{array}{c} s_{j}=\log S_{j} \end{array}$$ (3)

$$\begin{array}{c} a_{ij}=\log\left(\beta \right)+\log\left(P_{i1}\right)+\log\left(A_{ij}\right) \end{array}$$ (4)

$$\begin{array}{c} e_{jk}=\log\left(\frac{E_{jk}}{\beta }\right)+\log\left(\frac{P_{1k}}{P_{11}}\right) \end{array}$$ (5)

$$\begin{array}{c} p_{ik}=\log\left(\frac{P_{ik}}{P_{1k}}\right)+\log\left(\frac{P_{11}}{P_{i1}}\right) \end{array}$$ (6)

where *P*_1*k*_ is the detection probability of the species 1 (here, the weasel) in the status *k*, *P*_*i*1_ is the detection probability of dead animals of the species *i*, and *P*_11_ is the detection probability of dead weasels. It is then straightforward to show that, under the model (2), for all *i*,*j*,*k*:

$$\begin{array}{c} \log\lambda_{ijk}=s_{j}+a_{ij}+e_{jk}+p_{ik} \end{array}$$ (7)

This reparametrization has many interesting properties: (i) the Equation (7) defines a classical log-linear model that could be fitted using maximum likelihood [21]; (ii) the unknown parameters *a*_*ij*_, *e*_*j*2_ and *p*_*i*2_ are uniquely identifiable; (iii) the parameter *a*_*ij*_ corresponds to the relative density of the *i* ^th^ species in the *j* ^th^ SAR measured on a logarithmic scale, i.e., it corresponds to the desired density estimation, multiplied by an unknown constant *βP*_*i*1_ variable among species; (iv) the unknown parameter *e*_*j*2_ corresponds to the relative sampling effort—i.e., the sampling effort multiplied by an unknown constant *P*_*ik*_/(*βP*_11_)—of the living animals in the *j* ^th^ SAR measured on a logarithmic scale; (v) the parameter *e*_*j*1_ = log(*E*_*j*1_/*β*) = log*V*_*j*_ corresponds to the known sampling effort for the dead animals in the SAR *j* (this known effort was included in the model as an offset variable to allow the fit); (vi) the parameters *p*_*ik*_ are complicated functions of the detection probabilities. Note that our reparametrization implies the following identifiability constraints: *p*_*i*1_ = *p*_12_ = 0. Thus, only the unknown parameters *p*_*i*2_,*i* ≠ 1 were estimated.

### Accounting for spatial regularity and environmental filtering

The model developed in the previous section provided a way to estimate the relative densities of the different species of interest in the SARs, while accounting for unequal sampling effort among SARs and status, as well as different detectabilities for the different species and status. Thus, by assuming that the sampling effort was known in all SARs for the dead animals, it could have been possible to estimate the *I*×*J* log-relative densities *a*_*ij*_, the *J* log-relative sampling efforts *e*_*j*2_ for living animals, as well as the *I*−1 “noise” parameters *p*_*i*2_, by maximizing the log-likelihood [21]:

$$\begin{array}{c} \log ℒ=\sum_{i=1}^{I}\sum_{j=1}^{J}\sum_{k=1}^{K}N_{ijk}\left(a_{ij}+e_{jk}+p_{ik}\right)-\exp\left(a_{ij}+e_{jk}+p_{ik}\right) \end{array}$$ (8)

This is the classical approach to fit this type of log-linear model, available in every statistical software program.

However, at this point, we chose to introduce spatial and environmental knowledge into the modelling through constraints on the parameters, thus improving the precision of the estimates. On the one hand, it was likely that the species densities were spatially structured: because of spatial proximity, two neighboring SARs were probably characterized by more similar density profiles than two randomly drawn SARs. On the other hand, it was also likely that the species densities were affected by the environmental composition; two SARs with a similar environment were probably characterized by more similar density profiles than two SARs with highly differing conditions.

We chose to account for this spatial regularity and this environmental effect by maximizing a regularized log-likelihood [22, 23]. We used a ridge-type regularization method, i.e., a penalization of the likelihood ensuring a certain level of spatial and environmental regularity, where the differences between parameters are constrained to be small. Precisely, we considered the maximization of the following criteria:

$$\begin{array}{c} \log ℒ-\sum_{i=1}^{I}\sum_{j=1}^{J}\sum_{m=1}^{J}\nu \pi_{jm}{\left(a_{ij}-a_{im}\right)}^{2} \end{array}$$

with respect to the parameters *a*_*ij*_,*e*_*jk*_,*p*_*ik*_, where *π*_*jm*_ is a measure of “environmental and spatial proximities” between the SAR *j* and the SAR *m* (see below) and *ν* is a positive parameter that determines the strength of the penalty. It is clear from this equation that setting *ν* to a high value for a given species would strongly penalize the regularized likelihood if neighboring regions in the sense of the proximities *π*_*jm*_ were characterized by strongly different density estimates. Conversely, setting *ν* to zero would result in the classical maximum likelihood estimation.

We defined the proximities *π*_*jm*_ to take into account spatial and environmental proximities. We considered that two SARs belonging to the same large agricultural region were characterized by a more similar environment than two SARs belonging to differing large agricultural regions. We defined the spatial and environmental proximities in the following way: (i) *π*_*jm*_ = 1 when the SARs *j* and *m* are spatially adjacent and belong to the same large agricultural region, (ii) *π*_*jm*_ = 0.5 either when the SARs *j* and *m* are spatially adjacent but do not belong to the same large agricultural region (the most common case where *π*_*jm*_ = 0.5), or are not spatially adjacent but belong to the same large agricultural region (which is rarer, as 83% of the large agricultural regions contain at most two SARs), and (iii) *π*_*jm*_ = 0 otherwise. Note that a cross-validation approach indicated that a regularization based on proximities accounting for both spatial and environmental structure resulted in a much smaller prediction error than a regularization based on purely spatial proximities, thereby suggesting that our combination of space and environment was required for the density estimation (see S1 Appendix, section 7).

We selected the best value of the shrinkage parameter *ν* with a *R*−fold cross-validation approach (p. 241 in [22]: we have split the data into *R* parts, each part corresponding to one year of data collection (i.e. *R* = 4). For the *r* ^th^ part and for a given value of *ν*, we have fitted the model to the other *R*−1 parts of the data and calculated the prediction error of the fitted model when predicting the *r* ^th^ part of the data, using the negative log-likelihood of the predicted part of the data, i.e.,:

$$\begin{array}{ccc} Q^{\left(r\right)}\left(\nu \right) & = & \sum_{i=1}^{I}\sum_{j=1}^{J}\sum_{k=1}^{K}N_{ijk}^{\left(r\right)}\left\{s_{j}+a_{ij}^{\left(-r\right)}+e_{jk}^{\left(-r\right)}+p_{ik}^{\left(-r\right)}-\log\left(R-1\right)\right\}-\\ & & \exp\left\{s_{j}+a_{ij}^{\left(-r\right)}+e_{jk}^{\left(-r\right)}+p_{ik}^{\left(-r\right)}-\log\left(R-1\right)\right\} \end{array}$$ (9)

where the exponent (−*r*) indicates that the parameter has been estimated with the *r* ^th^ part of the data removed, and $N_{ijk}^{\left(r\right)}$ is the number of animals of the species *i* detected with status *k* in the *j* ^th^ SAR during the *r* ^th^ year. Note that the component log(*R*−1) appearing twice in the above formula allows for the prediction of the number $N_{ijk}^{\left(r\right)}$ of animals detected over only one year using the effort $e_{jk}^{\left(-r\right)}$ estimated over *R*−1 years. We calculated *Q* ^(*r*)^ for *r* = 1,2,…,*R* and combined the *R* estimates of prediction error with:

$$\begin{array}{c} Q\left(\nu \right)=\sum_{r=1}^{R}Q^{\left(r\right)}\left(\nu \right) \end{array}$$ (10)

We selected the value of *ν* for which the prediction error *Q*(*ν*) was the smallest (note that other criteria measuring the prediction error returned similar results; see S1 Appendix, section 4.1.1).

### Two possible measures of effort for the dead animals

We did not know exactly the number of kilometers traveled by the officers in each SAR during the study period. However, the SCSL database contained enough information to estimate it. We compared two alternative ways to estimate the number of kilometers traveled by the officers in each SAR during the study period (see also section 2.3 of S1 Appendix).

A first measure $V_{j}^{\left(1\right)}$ could be derived from the total number of kilometers traveled in a department (calculated with Equation 1) by supposing that the effort was uniformly distributed in the department. Under this assumption, we could estimate the number of kilometers traveled in each SAR by redistributing this total number across SARs as a function of their area. Thus, *S*_*j*_ being the area covered by the *j* ^th^ SAR, we approximated the number of kilometers $V_{j}^{\left(1\right)}$ traveled in each SAR with:

$$\begin{array}{c} V_{j}^{\left(1\right)}=T_{d}\times \frac{S_{j}}{\sum_{j^{'}\in D}S_{j^{'}}} \end{array}$$

where *D* is the set of SARs belonging to the department *d*.

We also considered another measure $V_{j}^{\left(2\right)}$, generated by a more complicated model allocating the total number of kilometers traveled by the officers of a department to the SARs of this department: this alternative model accounted for the fact that a given car generally concentrates its activity in a restricted area within the department (each brigade generally works on an informal “territory”). For each car, we calculated a barycenter of activity based on the mean location of the animals detected by this car. When pooled over all cars of the department, the distribution of the distances between the detected animals and these barycenters was log-normal with a mean and a standard deviation depending on the department. Therefore, for each department and each car in particular, we were able to estimate a two-dimensional function giving the probability density of presence of the car at each point. Averaging these densities over all cars, we obtained a function giving the probability density of presence of a car at each point in the department. After integration of this probability density function over a given SAR within a department, we could estimate the proportion of time spent by the cars of the department in the SAR. We could finally estimate the number of kilometers traveled by the cars of a department in a SAR by multiplying the total number of kilometers *T*_*d*_ with this proportion.

To identify the best measure of effort, we have compared the predictive efficiency of our model when the sampling effort of the dead was measured either with $V_{j}^{\left(1\right)}$ or with $V_{j}^{\left(2\right)}$. We used a cross-validation approach: we have considered separately the even years (2002, 2004) and the odd years (2003, 2005) of our study period; for each measure, we fitted a model using the data collected during the even years (using for each one the optimal value of *ν*, selected using the approach described in the previous section) and we measured the prediction error on the odd years, and conversely. The prediction error was measured using the criterion *Q* described in the previous section (see section 6 of S1 Appendix).

The model was fitted using a C program interfacing with the R software [24]. This program is available in the function penalizedmodel() of the package scsl (S1 Package). Moreover, we bootstrapped our data 100 times to allow for the calculation of the coefficients of variation for the relative density estimates.

### Ethics statement

The fieldwork has been made by 1500 professional officers working for the ONCFS. The data collection for the SCSL program was a part of their job, so that their written or verbal consent for this specific study was not required. These qualified officers were commissioned by the French Minister of Ecology, which allowed them to work with dead animals, according to the French legislation. The SCSL program was approved by the director of the ONCFS.

## Results

We fitted the penalized log-linear model developed in the previous section on the complete dataset, including the data concerning the common genet and the wildcat. Indeed, even if these two species were not of interest to us, including these data in the fit added precision to the estimation of the effort *e*_*j*2_ (these detections actually proved the presence of the officers at those places).

We first compared the two possible measures of sampling effort using cross-validation. The negative log-likelihood of the dataset estimated with cross-validation was much higher for the measure $V_{j}^{\left(2\right)}$ (*Q*_2_ = 5468) than for the measure $V_{j}^{\left(1\right)}$ (*Q*_1_ = 5240), indicating that the latter was characterized by the best predictive efficiency (see S1 Appendix, section 6, for further details). This result may be explained by the fact that the main role of the national wildlife protection officers is to ensure that the enforcement of the laws pertaining to the hunting and trapping of wild animals is as uniform as possible over a department, which suggests that their frequency of use of the SARs during the study period was proportional to their area. We therefore fitted our model using the measure $V_{j}^{\left(1\right)}$ of the sampling effort.

**Fig 2:**
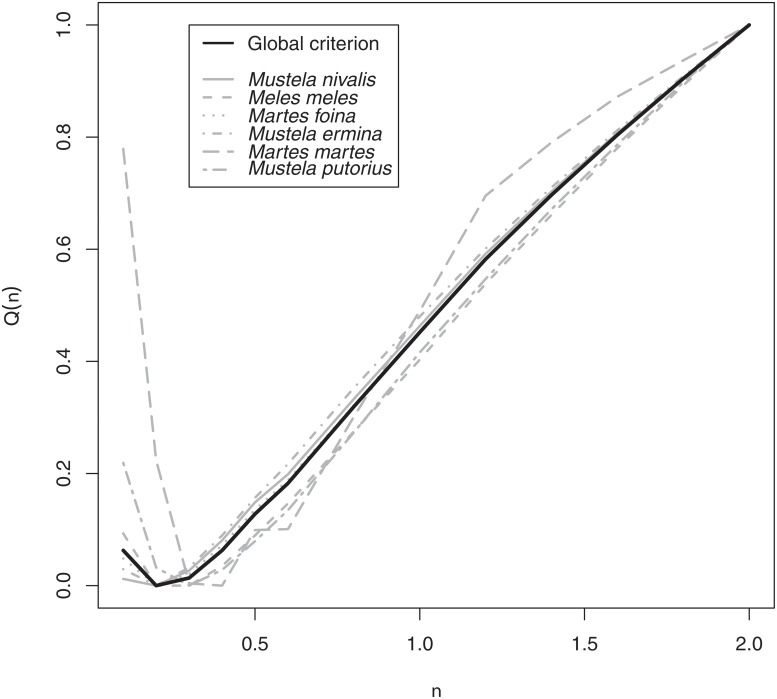
Value of the prediction error *Q*(*ν*) for different values of the penalty parameter *ν* calculated by the cross-validation approach. We also show the contributions *Q* ^*i*^(*ν*) of each species *i* to this prediction error. To make easier the visual interpretation of these results, the criteria *Q*(*ν*) and *Q* ^*i*^(*ν*) have been rescaled between 0 and 1 in the range of *ν* of interest.

**Fig 3:**
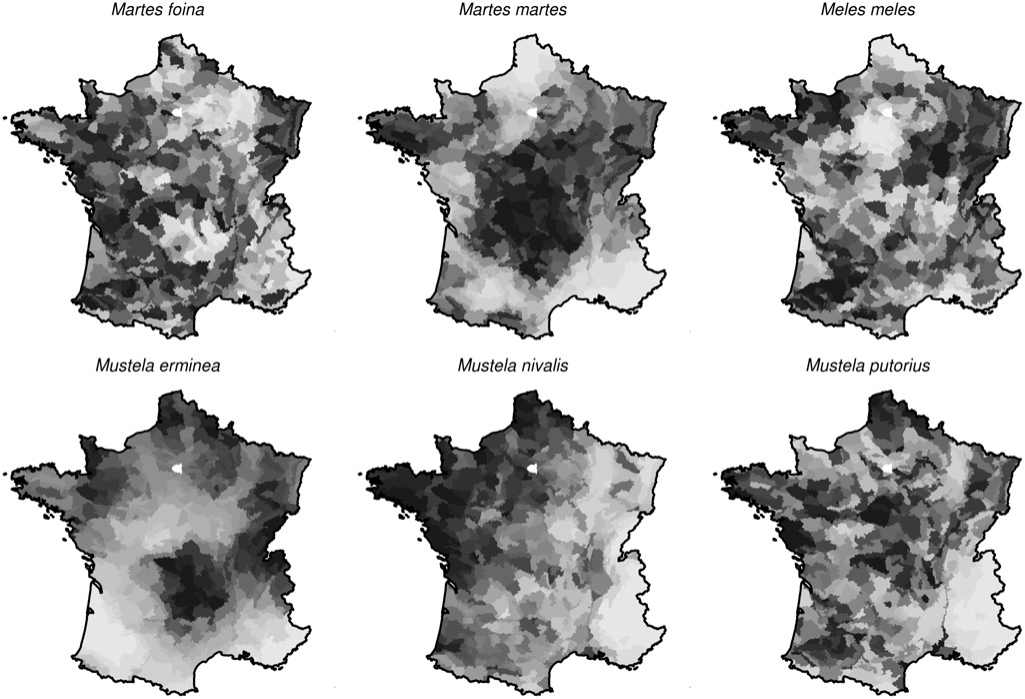
Relative density of the 6 *Mustelidae* species of interest as estimated by our model (darker areas correspond to higher densities).

For this model, the value of *ν* = 0.3 was the best choice to minimize the prediction error for all species (Fig. 2). This small penalty indicates that, at this scale, the spatial and environmental patterns of abundance are not very strong for most species (Fig. 3). The examination of the residuals did not reveal any problematic pattern, and the quality of the fit was satisfying. We also checked the absence of overdispersion in our fit by (i) checking that the standardized residuals were characterized by a constant variance when plotted as a function of the predicted values, and (ii) calculating the dispersion parameter associated to this model. This Pearson’s statistic was equal to *χ* ^2^ = 5962, for a number of degrees of freedom equal to *d* = 4913: the dispersion parameter was therefore estimated to be $\hat{h}=\chi^{2}/d=1.2$ (p. 175 in [25]), a value very close to 1 (see [26] for a discussion on this dispersion parameter and the identification of overdispersion). The absence of overdispersion in our model was therefore a reasonable hypothesis (see section 8 in S1 Appendix for further details).

The estimated densities (Fig. 3) for each species (dead and living animals) confirmed the known patterns of distribution, but improved our understanding at a smaller scale. The weasel appeared more abundant in the north-western part of France, and particularly in Brittany. The stoat was more abundant in the Massif Central, the mountainous area in the south-center of France, as well as in the eastern and northern parts of France. The badger and the stone marten were present in the whole country, with probable locally varying densities. The polecat was rare in the Alps and was more frequent in wettest areas of the country (e.g., Brittany). The pine marten was abundant in the Massif Central and in Brittany.

**Fig 4:**
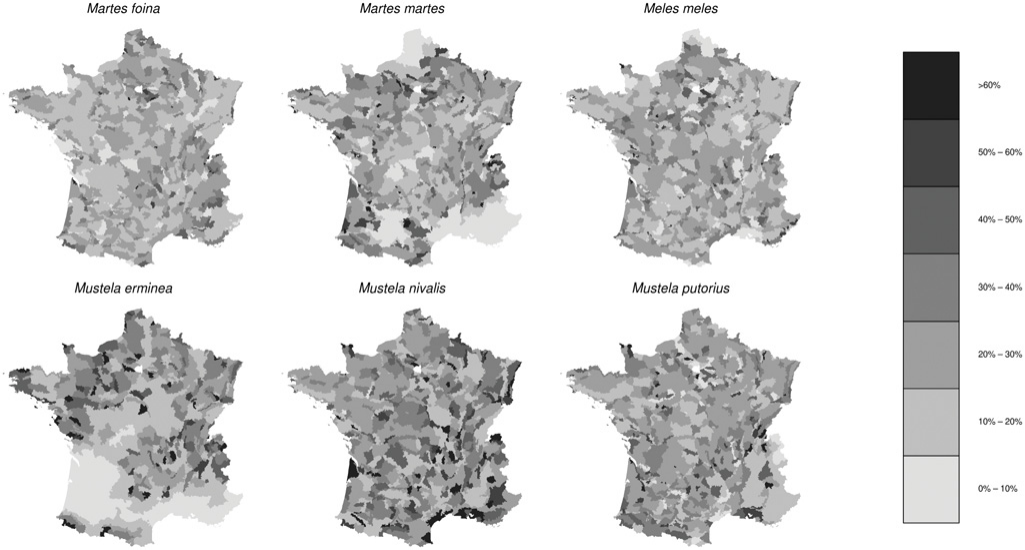
Coefficients of variation corresponding to the estimates of relative density for the 6 *Mustelidae* species of interest.

We also estimated the coefficients of variations associated with the relative density estimates for each species and each SAR (Fig. 4). In general, the coefficient of variation of the relative density estimate in a SAR was comprised between 15% and 35%.

## Discussion

**Fig 5:**
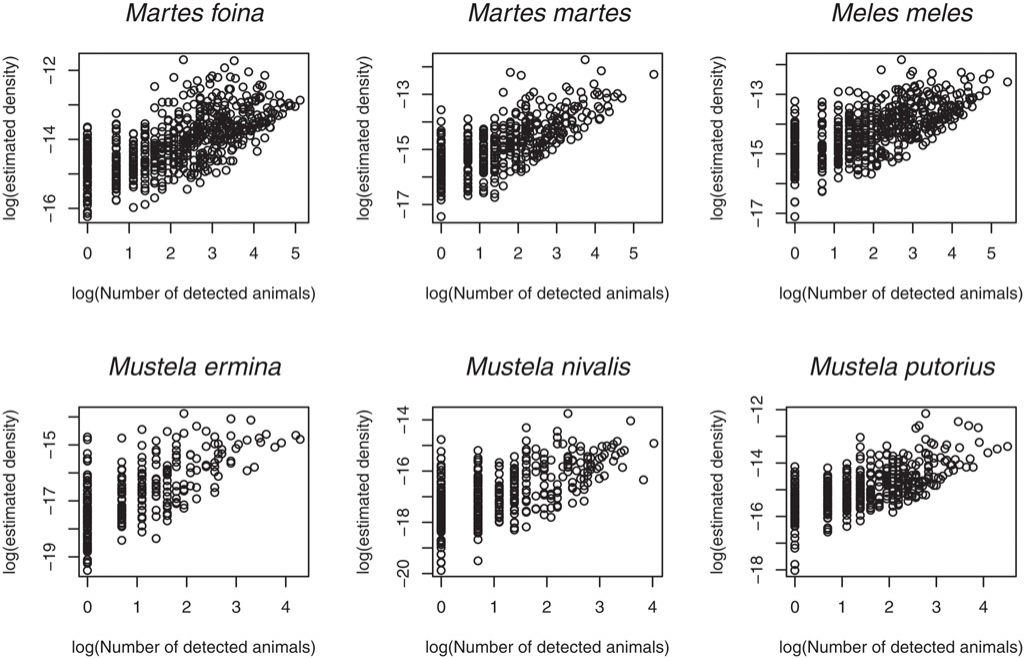
Relationship between the relative density as estimated by our model and the raw number of detection in each small agricultural region (SAR). These variables are showed on a log scale (we focused on SAR for which both the estimated density and the raw number of detections was greater than 0).

Until now, the data collected under the SCSL program were only used by the decision makers and private consultancy companies to derive distribution maps of the species from the raw numbers of detected animals. However, as many authors previously have noted, failing to account for unequal sampling effort as well as unequal detection probabilities precludes any density estimation [18]. We therefore developed a model taking into account an unequal observational effort across regions and detection status (dead/living animal), as well as a variable detection probability among species and status, to estimate the relative density of 6 *Mustelidae* species in each small agricultural region in France. Accounting for this effort in our model led to substantial differences between the estimated densities and the raw numbers of detected animals (see Fig. 5).

Our model supposes the absence of errors in species identification. Such errors can result into an overestimation of the abundance of a rare species if it is mistaken for a common species [27]. In this study, the observers were professionals trained to collect data for scientific programs; when they were uncertain of the species detected, they never reported the detection. Thus, to some extent, the identification uncertainty led to a smaller detection probability (see below). However, the fact that an officer was certain of the species identification does not mean that he actually identified the species correctly. An identification error was possible, especially when a living animal was detected, given that a close examination of the animal was in general not possible in this case. Identification errors were less likely when a dead animal was detected, which was the case of 74% of the badgers, polecats and martens. In addition, living animals were often detected by night, when visual identification was even more difficult. Such errors were also likely to vary among officers, especially with experience. Finally, identification errors were likely to depend on the species: they were expected to be rare for the well identifiable badgers. On the other hand, the stone marten and the pine marten are characterized by similar appearances and sizes and were more likely to be confused, though the differences of habitat used by the two species [28–30]—known to the officers – may have limited these confusions. The stoat and the weasel might also have been confused, but to a lesser extent since (i) these species were detected alive during the day, (ii) they are characterized by different sizes, and (iii) the prominent black tip of the tail of the stoat is highly visible during the day. Further studies are needed to account for this type of error in our model. One possible approach would be to model the probability of identification error using a dataset collected by a random sample of officers of the ONCFS with a double observer approach [27]. We could then fit our approach in a Bayesian framework, accounting for the uncertainty caused by identification errors using an informative prior distribution derived from this model of identification errors.

In this study, we supposed that the sampling effort of dead animals was proportional to the number of kilometers travelled by the officers. However, this is a very rough approximation of the actual effort, and it would be interesting to model this effort more precisely in a further study. Indeed, this sampling effort can be seen as the probability that a given animal *a* in a given region was detected as a dead animal by an officer. If we simplify the system by considering that all detected dead animals died as a result of roadkill, this probability is equal to the probability *p*_0_(*a*) that a given animal *a* was killed on the road, times the probability *p*_1_(*a*) that this killed animal was detected and reported by the officers. Our model did not account for all factors affecting these two probabilities, which may have resulted into biased estimates. On one hand, the probability *p*_0_(*a*) was probably not constant across SARs: indeed, roadkill is more frequent not only where the species is abundant, but also where the traffic load is important [31, 32]. Therefore, all other things being equal, the SARs with a more important traffic load were likely characterized by a larger number of roadkills. Not accounting for the difference of traffic load in our model may therefore have resulted into an overestimation of the relative abundance in the more urban SARs. On the other hand, this effect was mitigated by the fact that, within a given SAR, wildlife protection officers spent their time where the wildlife was, i.e in rural areas, driving along roads with light to moderate traffic. Thus, within a SAR, *p*_1_(*a*) was inversely related with *p*_0_(*a*), limiting this bias. Other factors may also have affected *p*_0_(*a*), such as curves in the roads and human disturbance [33], but how these small-scale factors affected the overall probability *p*_0_(*a*) in a given SAR is yet unclear, and further research is needed to model more precisely the sampling effort for the dead animals as a function of all variables affecting significantly *p*_0_(*a*) and *p*_1_(*a*).

Our approach also assumes that the density of a species along the roads of a SAR was proportional to its density in the places where the officers spent their time during static activities. Given that static activities were expected to be distributed uniformly over areas where the wildlife is present (i.e. over non-urban habitat types), this assumption could have been violated in two ways: (i) on a large scale, if the proportions of habitat types available in a given SAR did not correspond to the proportions of these habitat types along the roads, and (ii) on a small scale, if the local density of the species along a road was different from the density of interest in the habitat that it traversed. The point (i) was unlikely, because the roads are well distributed over the French territory, at a high density (1.77 km/km^2^, being on average comparable to road density in United Kingdom, Germany, Italy or Switzerland), and because there is no reason that roads would avoid certain habitat types. Concerning the point (ii), we note that the roads did not constitute a barrier to the movements of small carnivorous animals. These animals have indeed been shown to cross the roads in radio-tracking studies (Ruette, com. pers.). Of course, the fact that the roads did not constitute a barrier does not mean that they were used with the same frequency as the surrounding habitat. However, if there was a constant bias (e.g. if the density of a given species along the roads was always 20% smaller than the density in the surrounding habitat), this difference could be considered as part of the imperfect detectability of animals in our model, and would not result into biased estimates. On the other hand, if the bias was not constant (e.g. if the probability that an animal crossed the road was higher when the road was located in a forest), this would actually affect the estimates. Further research is needed to investigate how habitat selection by these small carnivorous species varies as a function of the distance to roads.

It is well known that the observer’s detection skills may vary and have problematic consequences in studies of species abundance [34]. Indeed, for a given number of traveled kilometers, a given officer observed the road and roadsides over only a fraction of these kilometers. This fraction was probably variable among officers. In our modeling context, a detection probability varying across observers was mathematically identical to a sampling effort varying across observers. Because a given officer team focused its activity on a restricted area within a department, it follows that the number of kilometers traveled by the team in that area was not necessarily exactly proportional to its sampling effort. For a given number of kilometers traveled in a region, a team with a larger detection probability was characterized by a larger “effort” than a team with a lower probability. However, we measured the sampling effort of the dead animals by summing the estimated individual efforts, i.e., a mathematical operation identical to averaging these efforts, up to a constant. Because there were on average 9 teams working on each SAR (interquartile range: 5—13), and each team was generally composed of two officers, averaging of the sampling effort over all teams working in a given SAR strongly decreased the heterogeneity in detection skills. Moreover, if we suppose that the detection skills and motivation of the officers were not spatially autocorrelated variables, the average sampling effort is expected to be proportional to the total number of kilometers in a given region. Given the homogeneity of the network of national wildlife protection officers in terms of duties and training, a spatial autocorrelation in their skills was not expected. However, we could not guarantee the absence of spatially structured motivation of the officers (i.e., the officers might have been more motivated to report a detection in some places than in others). It would therefore be interesting to study this heterogeneity based on a specific study, to assess the bias caused by this variation in space and time of the motivation.

**Fig 6:**
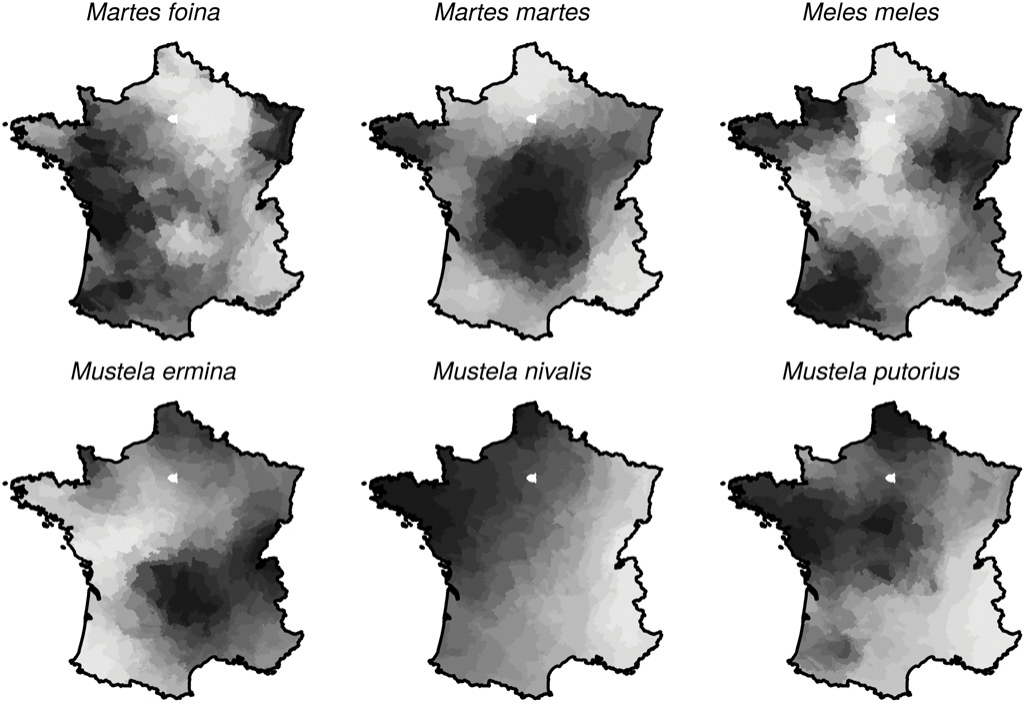
Spatial distribution of the 6 *Mustelidae* species of interest as estimated by a purely spatially regularized model, with a high penalty parameter *ν* = 20 (darker areas correspond to higher densities).

We used a ridge-type regularization to improve the prediction of our model by accounting for a spatial and environmental structure in the species density. Silverman [35] noted the following about another statistical method relying on a smoothing parameter: “the process of examining several plots of the data, all smoothed by different amounts, may well give more insight into the data than merely considering a single automatically produced curve”. Although this is not the main aim of our study, this remark applies to our modeling approach as well. Indeed, we replaced the spatial and environmental proximity metrics used in our regularization by a purely spatial proximity, with *π*_*jm*_ = 1 when SARs *j* and *m* are adjacent, and *π*_*jm*_ = 0 otherwise. Setting the parameter *ν* to a very large value (*ν* = 20) allowed us to identify the large scale distribution patterns of the species over the study area (Fig. 6). Although these maps are characterized by a larger bias and for this reason should not be used for management, they can be useful for exploration purposes. These maps indeed illustrated more clearly the broad presence areas identified in the Fig. 3.

In this paper, we have modeled the relative density of *Mustelidae* species using data collected over 4 years. As for all studies aiming at the abundance estimation, the density of a given species may have changed during the course of our study. Because the number of officers working for the program varies little from one year to the next, and because this period is short, it is acceptable to suppose a constant effort within a given SAR during the study period. Under this hypothesis, the model indeed allowed us to estimate the mean relative density of each species in each SAR, which was the quantity of interest for us. However, even if the study of those changes are not of interest for us, it could have been possible to study them with our modelling framework, just by replacing the factor “SAR” in our model by another factor combining the SARs and the years in the fit, leading to relative density estimates for the species in each SAR during each year. Note that we provide both the data and the functions required for this fit in the companion R package scsl (see S1 Package), so that the interested reader can try this “spatio-temporal” approach on our data. Note that if a monitoring of the relative density over time is carried out with this approach, it is important to prevent a decrease in the officers’ motivation with time (which would lead to an unmeasured decrease of the sampling effort, i.e. not strictly related to the number of kilometers). In our study, the data collected through the SCSL program are very simple data and quick to collect. They are useful to the officers themselves at the local scale (e.g. to build regional maps of distribution), which helps a lot to keep them motivated. We have also tried to motivate people through annual reports and regular communications, which helps them stay interested and updated in the project.

Finally, our modeling approach in itself has interesting implications. Indeed, this approach makes it possible to combine two datasets collected on the same population: (i) one dataset focusing on the dead animals collected with a supposed known sampling effort, and (ii) one dataset collected on the living animals with an unknown sampling effort. This ability of our modeling approach to combine information collected with different methods on the same population has interesting implications for the analysis of data collected in citizen sciences programs [18]. Indeed, our modeling framework could indeed be used to combine (i) a dataset collected using a known sampling design generally conceived by a scientific team and (ii) an opportunistic dataset obtained by crowdsourcing, collected by volunteers, generally without any knowledge of the sampling effort. Our approach would allow us to estimate the sampling effort for the opportunistic dataset, and correct the biases generated by the uncontrolled and unknown effort in such data. Preliminary results suggest that this approach would allow us to obtain unbiased and precise estimates of the relative densities of the studied species (Giraud et al. in prep.). This confirms the remark of MacKenzie et al. [36]: “in some situations, it may be appropriate to share or borrow information about population parameters for rare species from multiple data sources. The general concept is that by combining the data, where appropriate, more precise estimates of the parameters may be obtained.” Further work is now required to identify the conditions under which our approach could be used with data collected under citizen science programs.

## Supporting Information

S1 Appendix Details of the calculations carried out to fit the models. (PDF)

S1 Package R package containing the datasets and the functions. (GZ)
