# Supplementary material for: The Spatial Distribution of Mustelidae in France
Source: PLoS One. 2015 Mar 26;10(3):e0121689. doi: 10.1371/journal.pone.0121689 (PMC4374970; doi:10.1371/journal.pone.0121689)
Supplement: S1 Appendix — (PDF) [file pone.0121689.s001.pdf]

The spatial distribution of *Mustelidae* species in France: Appendix

Clément Calenge, Joël Chadoeuf, Christophe Giraud, Sylvie Huet
Romain Julliard, Pascal Monestiez, Jeremy Piffady, David Pinaud
Sandrine Ruette

Contents

1 Introduction: Aim of this document **3**

2 Data formating **4**

2.1 The data 4

2.2 Formatting the data 5

2.3 Two possible measures of effort 6

2.3.1 A sampling effort uniformly distributed within the department: measure $V_j^{(1)}$ 6

2.3.2 Another measure taking into account the officers behaviour: measure $V_j^{(2)}$ 7

3 The algorithms used for model fitting **12**

3.1 Steepest descent algorithm (`typealgo = 1`) 13

3.2 "Mixed" approach (`typealgo = 2`) 14

3.3 Quasi-Newton algorithm – BFGS (`typealgo = 3`) 15

4 Model fit conditional on the measure of effort $V_j^{(1)}$ **16**

4.1 Selection of the penalty parameter ν by cross-validation 16

4.1.1 Four possible criteria to measure the prediction error 16

4.1.2 Implementation of the cross-validation approach 17

4.2 Model fit with $\nu = 0.3$ 21

5 Model fit conditional on the measure of effort $V_j^{(2)}$ **24**

5.1 Selection of the penalty parameter ν by cross-validation 24

5.2 Model fit with $\nu = 0.3$ 28

6 Which effort measure is the best? **30**

6.1 Implementation of a cross-validation approach 30

6.2 Results 34

7 Is a purely spatial regularization a better choice to fit our model? **35**

7.1 Implementation of a cross-validation approach 35

7.2 Comparison of a purely spatial regularization with the spatial+environmental regularization 37

8 Model examination **41**

8.1 Model residuals and overdispersion 41

8.2 Goodness of fit 46

8.3 Coefficients of variation associated to the estimates 48

9 Addendum: purely spatial model **50**

1 Introduction: Aim of this document

In this document, we detail the calculations carried out to estimate the relative density of six species of *Mustelidae* in every small agricultural region (SAR) in France. We provide the data, the C code, and the R code to fully reproduce the analyses carried out for the paper. We also provide additional elements related to the model-fitting process. This appendix is written with Knitr (Xie, 2013), a system allowing to include R code in a LaTeX document (Lamport, 1994).

More precisely, in this appendix:

- We import, organize and format the data for the fit;
- We describe three available fitting algorithms;
- We fit a first model, using a measure of the sampling effort for the dead animals supposed to be constant within the department (i.e. the measure described in the paper). We use a cross-validation approach to select the optimal value for the penalty parameter;
- We fit another model, using an alternative measure of the sampling effort for the dead taking into account the officers behaviour. We select the optimal value for the penalty parameter using a cross-validation approach;
- We use another cross-validation approach to compare the two possible measures of sampling effort, and we show that the simplest one, i.e. the measure supposed to be constant within a department, is the best choice;
- We use the same type of cross-validation approach to compare the model based on a spatial and environmental regularization with a model based on a purely spatial regularization. We show that accounting for both the spatial and the environmental autocorrelation allows to reduce the prediction error significantly;
- We study the residuals and the goodness of fit of the final model;
- We estimate the coefficient of variations associated to the estimated relative densities using a bootstrap approach.
- We illustrate the use of this modelling approach for exploratory purposes (i.e. to identify the main distribution patterns in space), as pointed out at the end of the discussion of the paper.

We also provide an R package named `scsl` containing the datasets used in the paper as well as the R functions implementing the models. Because the calculations carried out in this paper are often cumbersome, we have programmed the relevant algorithms in C. The R functions available in the package are just interfacing the C procedures. Thus, the model used in the paper is described on the help page of the function `penalizedmodel()`. The function `crossvalidation()` can be used to identify the optimal value of the penalty. The function `bootstrapcisstat()` can be used to estimate the standard errors of the estimates with a bootstrap approach.

The data collected under the small carnivorous species logbook program are available in the following datasets: (i) the dataframe `scsl` contains the data related to the detected animals, (ii) the dataframe `carsscsl` contains the data related to the cars working in this program, (iii) the dataset `department` contains a map of the French department, and (iv) the dataset `SAR` contains a map of the small agricultural regions in the department.

We first describe how to install the package `scsl` provided with this appendix. This package contains source C code, and this code needs to be compiled at the install time. Therefore, the operating system of the reader needs a

C compiler. Such compilers are usually available on Linux. On Mac OS X, the developers tools should be installed (they come in the Mac OS X installation DVD). However, there is no C compiler available by default on Windows. Windows users will need to install the Rtools to reproduce the calculations of this document. These tools can be downloaded at the following URL: http://cran.r-project.org/bin/windows/Rtools/. Select the suitable version corresponding to the version of the R software used by the reader. Then install the program, and **select the checkbox "add to the variable PATH"** during the installation process.

Then, the reader will need to install the dependencies (i.e. the packages needed to reproduce the analyses):

```
install.packages(c("sp", "maptools", "ade4", "spdep",
    "Hmisc"), dependencies = TRUE)
```

Finally, the reader should place the package `scsl` in his working directory, and install the package `scsl`, using the following command line:

```
install.packages("scsl_1.0.tar.gz", repos = NULL, type = "source")
```

We can finally load the package `scsl`:

```
library(scsl)
```

2 Data formating

2.1 The data

We first load the data required for the analyses. The datasets `SAR` and `department` store the maps of small agricultural regions and departments respectively (objects of class `"SpatialPolygonsDataFrame"`):

```
data(department)
data(SAR)
```

We can plot these spatial data, and thereby reproduce fig. 1B of the paper:

```
plot(SAR, border = "grey")
plot(department, add = TRUE, lwd = 2)
```

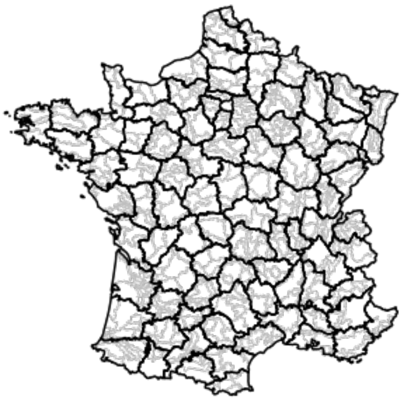

We also load the datasets `scsldetect`, which contains the information on the detected animals, and `carsscsl`, which contains the information on the cars:

```
data(scsldetect)
head(scsldetect)

##   Department Year      Species Status   SAR Car
## 1         01 2002  MartesFoina      D 01446   1
## 2         01 2002  MartesFoina      D 01446   1
## 3         01 2002  MartesFoina      L 01446   1
## 4         01 2002   MelesMeles      D 01446   1
## 5         01 2002 MartesMartes      D 01446   1
## 6         01 2002  MartesFoina      L 01446   1

data(carsscsl)
head(carsscsl)

##   Car Year Department    Km
## 1   1 2002         01    NA
## 2   1 2003         01    NA
## 3   2 2002         01    NA
## 4   3 2002         01 23411
## 5   4 2002         01    NA
## 6   5 2002         01 21366
```

Additional information on these datasets is available on the help pages of these datasets. For example, for information about the variable names in the data.frame `scsldetect`, type:

```
help("scsldetect")
```

2.2 Formatting the data

First, we prepare the data for the model fit. We will fit the model with the function `penalizedmodel`. The format required for this fit is described on the help page of this function:

```
help("penalizedmodel")
```

We build the data.frame required for the analysis below. This data.frame contains the number of detections for every combination of the factors species, small agricultural region (SAR) and status. It also contains the area of the SARs:

```
dfdata <- do.call("rbind", lapply(unique(scsldetect$Species),
    function(i) {
        ## we keep only the observations of each species
        scsle <- scsldetect[scsldetect$Species == i,
            ]
        ## For the dead as for living animals
        do.call("rbind", lapply(c("D", "L"), function(k) {
            ## and for each sar
            do.call("rbind", lapply(as.character(SAR@data$codeSAR),
                function(sarr) {
                  ## Return the results
                  data.frame(Nijk = nrow(scsle[as.character(scsle$SAR) ==
                    sarr & scsle$Status == k, ]), Sp = i,
```

```
                    Sta = k, SAR = sarr)
                })) 
        }))
    }))

## calculation of the SAR area
area <- sapply(as.character(dfdata$SAR), function(x) {
    SAR@data$Area[as.character(SAR@data$codeSAR) ==
        x]
})

## The area is added to the data.frame
dfdata <- data.frame(dfdata, Sj = area)
```

We also calculated the environmental and spatial proximities π_{jm}, for every pair of SARs j and m. Recall that we defined these proximities in the following way: (i) $\pi_{jm} = 1$ when the SARs j and m are spatially adjacent and belong to the same large agricultural region, (ii) $\pi_{jm} = 0.5$ when the SARs j and m belong to the same large agricultural region but are not spatially adjacent, or are spatially adjacent but do not belong to the same large agricultural region, and (iii) $\pi_{jm} = 0$ otherwise. We calculated the $J \times J$ proximity matrix below:

```
## First step: matrix storing the neighbouring
## relationships Makes use of the functions of spdep
## and ade4
library(spdep)
library(ade4)
pg <- neig2mat(nb2neig(poly2nb(SAR)))

## Second step: identify the large agricultural
## region to which each SAR belong. The SAR code
## stores this information. Indeed, this code is:
## Department (two digits) + large agric. region (3
## digits) We get the 3 last digits
larcode <- substr(SAR@data$codeSAR, 3, 5)
## Calculates a matrix storing, for each SAR, if it
## belongs to the same large agric region as the
## other SARs
aprgr <- sapply(1:nrow(SAR), function(i) as.numeric(larcode ==
    larcode[i]))
## The diagonal is equal to 0
diag(aprgr) <- 0
## Calculate the proximity matrix
prx <- (pg + aprgr)/2
```

2.3 Two possible measures of effort

As indicated in the paper, we considered two alternative measures of observational effort. Because we know the number of kilometers traveled by each car (stored in the data.frame `carsscsl`), we know the observational effort at the scale of the department. However, we need a way to attribute a sampling effort at a smaller scale, i.e. the SAR scale.

2.3.1 A sampling effort uniformly distributed within the department: measure $V_j^{(1)}$

The first possible measure that we considered is the measure described in the main paper. We supposed that the sampling effort was uniformly distributed within a department. In other words, the sampling effort of a given SAR

was calculated with:

$$V_j^{(1)} = B_d \times \frac{1}{R_d}\left(\sum_{c=1}^{R_d} k_c\right) \times \frac{S_j}{\sum_{j' \in D} S_{j'}}$$

where B_d is the total number of logbooks available in the department d during the study period, R_d is the number of logbooks available in this department for which the yearly distance traveled was recorded, k_c is the number of kilometers traveled by the car c, S_j is the area of the SAR j, and D is the set of SARs belonging to the department d (see the main paper). We calculated this effort for each SAR below:

```
## Calculation of the observational effort
Vj <- sapply(SAR@data$codeSAR, function(y) {
    ## Identifies the department code from the SAR code
    ## (the first two numbers of the SAR code are the
    ## department code)
    x <- substr(as.character(y), 1, 2)

    ## Departments of the south of the Parisian region
    ## are pooled together
    if (any(c("91", "95") == x))
        x <- "78"

    ## Calculation of the total sampling effort in each
    ## department, allocated to each SAR as a function
    ## of the area
    length(carsscsl$Car[as.character(carsscsl$Department) ==
        x]) * mean(na.omit(carsscsl$Km[as.character(carsscsl$Department) ==
        x]))/department@data$Area[department@data$Code ==
        x]
})

## The variable 'effort'
Vj1 <- sapply(as.character(dfdata$SAR), function(x) Vj[SAR@data$codeSAR ==
    x])
```

This sampling effort is stored in the vector `Vj1`.

2.3.2 Another measure taking into account the officers behaviour: measure $V_j^{(2)}$

We also considered an alternative measure $V_j^{(2)}$ of effort. This alternative measure accounted for the fact that a given team of officers generally works on a restricted area within the department. For a given car, the activity is therefore not homogeneously distributed within the department. We have therefore measured the sampling effort for each SAR using the following approach:

- For each car with at least 5 detected animals, we have calculated the center of gravity of the observations (all years pooled), as well as the distance between the observations and this center of gravity;

- We have associated a center of activity to each car. When the car had detected at least 5 animals, the center of activity corresponded to the center of gravity of these observations. Otherwise, this center of activity corresponded to the center of gravity of another car randomly sampled within the same department,

- The distribution of the distances between the animals detected by a car and its center of gravity was log-normal with a mean and a standard deviation depending on the department. Therefore, under this hypothesis and knowing the center of activity of every car, we could estimate a two-dimensional function giving the probability density of presence of each car at each point in every department.

- From this two-dimensional function, we estimated the proportion of the time spend by each car in every SAR of the department. Moreover, we generally knew the total number of kilometers traveled by a given car a given year (when this number was missing, we randomly sampled this value in the set of available mileages in the department). We multiplied this number of kilometers with the proportion of the activity time spent by this car in a given SAR to obtain the number of kilometers traveled in the SAR;

- Finally, we have divided this number of kilometers by the area of the SAR. This standardized number of kilometers is our measure of effort.

We calculated this alternative measure of effort below:

```
## The department code is coded as a factor
department@data$Code <- factor(department@data$Code)

## List of the SAR per department
lideptsar <- lapply(levels(department@data$Code), function(x) {
    as.character(SAR@data$codeSAR)[as.character(SAR@data$Department) ==
        x]
})

## Note that the departments 91, 95 and 78 are
## grouped and coded 78
ee <- unlist(lideptsar[levels(department@data$Code) %in%
    c("78", "91", "95")])
lideptsar[levels(department@data$Code) == "78"][[1]] <- ee

lideptsar <- lideptsar[!levels(department@data$Code) %in%
    c("91", "95")]

## coordinates associated to the observations
coop <- coordinates(SAR)
dfcoop <- data.frame(as.data.frame(coop), sar = SAR@data$codeSAR)
wh <- lapply(as.character(scsldetect$SAR), function(x) {
    which(as.character(dfcoop$sar) == x)
})
cooobs <- dfcoop[unlist(wh), 1:2]

## List of the barycenters of the cars for each
## department
libar <- lapply(as.character(department@data$Code),
    function(x) {
        ## Show the progression
        cat(round(100 * which(as.character(department@data$Code) ==
            x)/nrow(department@data)), "%\r")
        ## The cars working in the department
        cars2 <- unique(as.character(carsscsl$Car[as.character(carsscsl$Department) ==
            x]))
        ## For each car
        resu <- do.call("rbind", lapply(cars2, function(y) {
            ## Identifies the coordinates of the animals
            ## detected by this car
            co <- cooobs[as.character(scsldetect$Car) ==
                y, ]
            ## If the car has detected at least animals
            if (nrow(co) > 4) {
                ## The barycenter of these observations
```

```
                bar <- unlist(apply(co, 2, mean))
                ## Return the cars and the coordinates of the
                ## barycenter
                return(data.frame(Car = as.character(x),
                  xbar = bar[1], ybar = bar[2]))
            } else {
                return(NULL)
            }
        }))
        return(resu)
    })


## Remove the few barycenter outside the departments
libar <- lapply(1:(length(libar) - 2), function(i) {
    ## for each department, get the list of barycenter
    x <- libar[[i]]
    ## transform it as a spatialPointsDataFrame
    coordinates(x) <- c("xbar", "ybar")
    ## get the code of the department
    dep <- as.character(department@data$Code)[i]
    ## get the list of SARs of this department
    listsar <- lideptsar[[i]]
    ## get the maps of these SARs
    sartt <- SAR[as.character(SAR@data$codeSAR) %in%
        listsar, ]
    ## overlay the barycenter on these sar
    ov <- over(x, sartt)
    ## and remove the cars with a barycenter outside the
    ## department
    libar[[i]][!is.na(ov[, 1]), ]
})


## List of the means and standard deviations of the
## log-distances between the observations and the
## barycenter
lidist <- lapply(as.character(department@data$Code),
    function(x) {
        ## Show the progression
        cat(round(100 * which(as.character(department@data$Code) ==
            x)/nrow(department@data)), "%\r")
        ## The cars working in the department
        cars2 <- unique(as.character(carsscsl$Car[as.character(carsscsl$Department) ==
            x]))
        ## For each car
        resu <- unlist(lapply(cars2, function(x) {
            ## Identifies the coordinates of the animals
            ## detected by this car
            co <- cooobs[as.character(scsldetect$Car) ==
                x, ]
            ## If the car has detected more than 4 animals
            if (nrow(co) > 4) {
                ## The barycenter of these observations
                bar <- unlist(apply(co, 2, mean))
                ## Centers the observations
                centr <- t(t(as.matrix(co)) - bar)
```

```
                ## Calculate the log-distance between the
                ## observations and the barycenter
                lo <- log(sqrt(apply(centr, 1, function(x) sum(x^2))))
                return(lo)
            } else {
                return(NULL)
            }
        }))
        ## removes the missing values
        resu <- resu[!is.na(resu)]
        resu <- resu[resu > -1e+09]
        ## Calculates the mean and sd of the log distance
        if (!is.null(resu)) {
            return(c(mean(resu), sd(resu)))
        } else {
            return(c(NULL, NULL))
        }
    })


## list of the number of kilometers available for
## each department
likm <- lapply(as.character(department@data$Code),
    function(x) {
        cars2 <- carsscsl[as.character(carsscsl$Department) ==
            x, ]
        cars2 <- cars2$Km[!is.na(cars2$Km)]
        return(cars2)
    })


## For each car, we associate the barycenter, the
## parameters of the distribution of log-distances,
## and the number of kilometers travelled for each
## year
res <- as.data.frame(do.call("rbind", lapply(1:nrow(carsscsl),
    function(i) {
        ## Identifies the barycenter
        ee <- as.character(department@data$Code) ==
            as.character(carsscsl$Department[i])
        de <- libar[ee][[1]]
        ba <- de[as.character(de$Car) == as.character(carsscsl$Car[i]),
            2:3]
        ## If there are not a number of detected animals
        ## large enough to allow the calculation of the
        ## barycenter
        if (nrow(ba) == 0) {
            ## Sample it among the other barycenter
            ba <- de[sample(1:nrow(de), 1), 2:3]
        }
        ## The parameters of the log-distance distribution
        padi <- lidist[as.character(department@data$Code) ==
            as.character(carsscsl$Department[i])][[1]]
        ## Number of kilometers
        km <- carsscsl$Km[i]
        ## if missing
        if (is.na(km)) {
```

```
            ## sample it among the available kilometers
            lik <- likm[as.character(department@data$Code) ==
                as.character(carsscsl$Department[i])][[1]]
            km <- sample(lik, 1)
        }
        ## Return the barycenter, the parameters of the
        ## log-distance distribution, and the number of
        ## kilometers
        return(c(unlist(ba), unlist(padi), km))
    })))


## Then, for each car, we generate random locations
## according to the distribution of log-distances.
## For each car, we identify the SARs where these
## locations are falling, we calculate the
## proportion of locations in each SAR, and we
## redistribute the kilometers in the SARs
## accordingly The result is a list with one element
## per car, each element corresponding to a vector
## with as many values as there are SARs in the
## department, each value corresponding to the
## estimated number of kilometers travelled in the
## SAR
effortpercar <- lapply(1:nrow(carsscsl), function(i) {
    ## Progression of the calculation
    cat(i/nrow(carsscsl), "%\r")
    ## list of SARs of the department where this car is
    ## traveling
    listofsars <- lideptsar[as.character(department@data$Code) ==
        as.character(carsscsl$Department[i])][[1]]
    ## map of the sars in this department
    sart <- SAR[as.character(SAR@data$codeSAR) %in%
        listofsars, ]
    ## We randomly draw 10000 points from a normal
    ## distribution with parameters corresponding to the
    ## estimated parameters for the log-distance and exp
    ## of it, to calculate distances
    dis <- exp(rnorm(10000, res[i, 3], res[i, 4]))
    ## We randomly draw angles from a circular uniform
    ## distribution
    ang <- runif(10000, 0, 2 * pi)
    ## And we generate the locations of virtual
    ## presences relative to the barycenter
    xc <- dis * cos(ang)
    yc <- dis * sin(ang)
    ## We add the position of the barycenter to have
    ## their coordinates
    xc <- xc + res[i, 1]
    yc <- yc + res[i, 2]
    ## conversion to a SpatialPoints object
    df <- data.frame(x = xc, y = yc)
    coordinates(df) <- c("x", "y")
    ## spatial join to identify the SAR where these
    ## presences are
    ov <- over(df, sart)
    df <- df[!is.na(ov[, 1]), ]
```

```
    saro <- as.character(ov$codeSAR[!is.na(ov[, 1])])
    ## proportion of presences in each SAR
    prop <- sapply(listofsars, function(x) length(saro[saro ==
        x])/length(saro))
    ## We redistribute the number of kilometers
    ## accordingly
    prop * res[i, 5]
})


## Construction of a list giving a new SAR code
## corresponding to each vector element in the above
## list (starting from 0)
linumsar <- lapply(effortpercar, function(x) {
    as.integer(sapply(names(x), function(y) {
        as.numeric(SAR@data$codeSAR)[as.character(SAR@data$codeSAR) ==
            y]
    }) - 1)
})

## Use the C function Calculeffort to calculate the
## average effort over each SAR This internal
## function returns a vector of length J (where J is
## the number of SARs) containing the number of
## kilometers travelled. There is no 'interface' R
## function for this C function. We rely on the call
## of the C function available in the dll of the
## package scsl.
Vj2 <- .Call("Calculeffort", effortpercar, linumsar,
    as.integer(rep(1, length(effortpercar))), as.double(SAR@data$Area),
    as.integer(nrow(SAR)), PACKAGE = "scsl")
```

This sampling effort is stored in the vector `Vj2`. We now have all the elements needed to implement our modelling approach.

3 The algorithms used for model fitting

Because our modeling approach is cumbersome to implement, we have chosen to program it with the C language after prototyping in the R language. We have considered three different ways to program this model. Indeed, because we wanted to use this approach intensively (cross-validation, bootstrap), the speed comparison allowed us to choose the fastest program. Moreover, the fact that the three programs returned the same results indicated that we did not made any programming error.

The speed comparison favoured the BFGS approach (see below), which we used in the rest of this document. We nevertheless also describe the two other available algorithms in this section. Indeed, all three optimization approaches are implemented in the function `penalizedmodel` and are therefore available to the reader. As noted on the help page of this function, the type of algorithm can be chosen by setting the option `typealgo` in the list passed to the parameter `options` of this function:

```
typealgo The type of algorithm used for the fit: value 1 =
     steepest descent; value 2 = mixed approach; value 3 = BFGS
     approach; value 4: approach consisting to use a steepest
     descent approach until a convergence evaluated with a
     threshold equal to ‘10^-6’, then a BFGS to reach convergence
     for the threshold defined with ‘stopCrit’. Default to 3.
```

3.1 Steepest descent algorithm (`typealgo = 1`)

Recall from the main text that minus the log-likelihood of the model has the following form:

$$-\log\mathcal{L} = \lambda_{ijk} - N_{ijk}\log\lambda_{ijk}$$

We use the same notations as in the main text here. Adding the regularization constraint, the penalized negative log-likelihood is:

$$-\log\mathcal{L} + P = \lambda_{ijk} - N_{ijk}\log\lambda_{ijk} + \sum_{i=1}^{I}\sum_{j=1}^{J}\sum_{m=1}^{J}\nu\pi_{jm}(a_{ij}-a_{im})^2$$

Let θ be the vector containing the unknown parameters a_{ij}, e_{j2} et p_{i2}. Let $C(\theta) = -\log\mathcal{L}+P$ be the criterion being minimized by the fit. The gradient vector $\nabla C(\theta)$ associated to this penalized negative log-likelihood is equal to:

$$\frac{\partial C(\theta)}{\partial a_{ij}} = \sum_k \left(\lambda_{ijk} - N_{ijk}\right) + 2\nu\sum_m \pi_{jm}(a_{ij}-a_{im}) \quad (1)$$

$$\frac{\partial C(\theta)}{\partial e_{j2}} = \sum_i (\lambda_{ij2} - N_{ij2}) \quad (2)$$

$$\frac{\partial C(\theta)}{\partial p_{i2}} = \sum_j (\lambda_{ij2} - N_{ij2}) \quad (3)$$

We develop the criterion $C(\theta)$ with a second order Taylor expansion in the neighborhood of θ':

$$C(\theta) = C(\theta') + \nabla C(\theta')(\theta-\theta') - \frac{\nabla^2 C(\theta')}{2}(\theta-\theta')^2$$

which can also be written:

$$C(\theta - h\nabla C(\theta)) = C(\theta) - h||\nabla C(\theta)||^2 + \frac{h}{2}\nabla C(\theta)^T\nabla^2 C(\theta')\nabla C(\theta')$$

Because this criterion is convex in θ, it follows that the last element of this equation is greater than 0:

$$\frac{h}{2}\nabla C(\theta)^T\nabla^2 C(\theta')\nabla C(\theta') \geq 0$$

and, if h is small, then:

$$C(\theta - h\nabla C(\theta)) \approx C(\theta) - h||\nabla C(\theta)||^2$$

In other words, subtracting $h\nabla C(\theta)$ to every value of θ allows to reduce the value of this criterion, when h is small. This suggests a means to find a value of θ that minimizes this criterion:

1. We select an inital value for the vector θ_1, a small value for h (we have chosen $h = 10$), and a threshold s below which we consider that the algorithm has converged ($s = 10^{-8}$).
2. We calculate $C_1 = C(\theta_1)$
3. We update the vector $\theta_2 = \theta_1 - h\frac{\nabla C(\theta_1)}{||\nabla C(\theta_1)||}$
4. We calculate $C_2 = C(\theta_2))$
5. If $(C_1 - C_2)/|C_1| > s$, this indicates that the algorithm has not converged yet. We set $\theta_1 = \theta_2$, $C_1 = C_2$, and we start the procedure again at step 3.
6. If $C_2 > C_1$, this means that the value of h is too large. We set $h = h/2$ and we start again the procedure at the step 3.

7. If $(C_1 - C_2)/|C_1| \leq s$, this means that the algorithm has converged. The value of θ which minimized the criterion is $\theta = \theta_2$.

This algorithm is used very commonly to find a solution for problems of this type (*steepest descent algorithm*, cf. NOCEDAL et WRIGHT, 2006, p. 21).

3.2 "Mixed" approach (`typealgo = 2`)

We have implemented another approach, which consists to update the parameters e_{j2}, p_{i2} and a_{ij} in turn. Indeed, we have developed the equation of the gradient vector associated to the penalized negative log-likelihood in the previous section. The criterion to be minimized being convex, this gradient vector takes a value equal to zero for the solution θ of the problem. Then we can find the solution by using the following algorithm:

1. Initialization: Definition of θ_1 containing the starting values for the parameters a_{ij}, e_{j2} et p_{i2}. We set them equal to the estimations of these parameters under the classical non-penalized maximum likelihood. We define a small value for h ($h = 10$), an a threshold s below which we consider that the algorithm has converged ($s = 10^{-8}$).

2. We calculate the criterion for the current values of the parameters: $C_1 = C(\theta_1)$

3. We update the parameters e_{j2}. From equation (2), we can update these parameters conditionally on the values a_{ij} and p_{i2}. Indeed, the value of e_{j2} that minimizes the penalized negative log-likelihood conditionally on these parameters can be calculated exactly, noting that the first derivative of the log-likelihood is equal to zero for the solution. Then:

$$\sum_i (S_j \exp(a_{ij} + e_{j2} + p_{i2}) - N_{ij2}) = 0$$

$$e_{j2} = \log \frac{\sum_i N_{ij2}}{\sum_i S_j \exp(a_{ij} + p_{i2})}$$

4. We update the parameters p_{i2}. Similarly, the value of p_{i2} minimizing the criterion conditionally on the values of a_{ij} et e_{j2} can be calculated exactly, noting that the first derivative of the log-likelihood is equal to zero at the solution. Then:

$$\sum_j (S_j \exp(a_{ij} + e_{j2} + p_{i2}) - N_{ij2}) = 0$$

$$p_{i2} = \log \frac{\sum_j N_{ij2}}{\sum_j S_j \exp(a_{ij} + e_{j2})}$$

   And we define $p_{12} = 0$;

5. We update the parameters a_{ij}. Contrarily to the two previous parameters, we cannot find analytically an exact solution to this minimization problem. We therefore use a *steepest descent* algorithm (similar to the algorithm developed in the previous section). More precisely, for each a_{ij}, we calculate the value of the first derivative of the penalized log-likelihood, given in the equation 1:

$$d = \sum_k (\lambda_{ijk} - N_{ijk}) + 2\nu \sum_l \pi_{ijl}(a_{ij} - a_{il})$$

   and we update the value de a_{ij} by defining the new value equal to $a_{ij} - hd$.

6. The updated values are stored in the vector θ_2. We calculate the penalized negative log-likelihood associated to this updated vector θ_2: $C_2 = C(\theta_2)$.

7. If $(C_1 - C_2)/|C_1| > s$, this means that the algorithm has not converged yet. We set $\theta_1 = \theta_2$, $C_1 = C_2$, and we start the procedure again at step 3.

8. If $C_2 > C_1$, this means that the value of h is too large. We set $\theta_1 = \theta_2$, et $h = h/2$ and we start the procedure again at step 3.

9. If $(C_1 - C_2)/|C_1| \leq s$, this means that the algorithm has converged. The value of θ minimizing this criterion is $\theta = \theta_2$.

3.3 Quasi-Newton algorithm – BFGS (`typealgo = 3`)

The quasi-Newton algorithm is a common approach identify the solution to such optimization problems (Nocedal et Wright, 2006, p. 22 and following). This algorithm can be simply understood by considering the Taylor expansion of the penalized negative log-likelihood, described in the previous sections:

$$C(\theta + \mathbf{p}) \approx m_k(\mathbf{p}) = C(\theta) + \mathbf{p}^T \nabla C(\theta) + \frac{1}{2}\mathbf{p}^T \nabla^2 C(\theta)\mathbf{p}$$

If we suppose that $\nabla^2 C(\theta)$ is positive definite (which is actually the case, given the convexity of the criterion to be minimized), then the solution is found by searching the value of $\mathbf{p}$ that minimizes this function. We can find this solution by identifying the value of $\mathbf{p}$ that cancels the value of $m_k(\mathbf{p})$. This solution is:

$$\mathbf{p}_k^N = -\left(\nabla^2 C(\theta)\right)^{-1} \nabla C(\theta)$$

The solution direction $\mathbf{p}_k^N$ is named *Newton direction*, and is generally different from the steepest descent direction. This direction can be used to find the solution immediately when the difference between the criterion and its quadratic model (i.e., the Taylor expansion) is equal to zero. When this difference is small, a small number of iterations of this algorithm allows to find the solution. One great advantage of the Newton direction in comparison to the steepest descent direction is that there is no need to set a step size.

The main drawback of this approach is that it requires the calculation of the Hessian matrix $\nabla^2 C(\theta)$, which can be cumbersome, in particular when the number of parameters increases. For this reason, we use another direction, the *Quasi-Newton* direction, which relies on an approximation B_k of the Hessian matrix. This approximation is updated after each iteration k of the algorithm. The most common formula to calculate this matrix is the BFGS formula (named after its inventors: Broyden, Fletcher, Goldfarb, and Shanno):

$$B_{k+1} = B_k - \frac{B_k s_k s_k^T B_k}{s_k^T B_k s_k} + \frac{y_k y_k^T}{y_k^T s_k}$$

with:

$$y_k = \nabla C(\theta_{k+1}) - \nabla C(\theta_k)$$
$$s_k = \theta_{k+1}\theta_k$$

For additional details, see Nocedal et Wright (2006, p.22).

This approach is implemented in the R function `optim`. This function relies on a C procedure available to C programmers through the API of the R software (see `http://cran.r-project.org/doc/manuals/R-exts.html`, section 6.8). Our function `penalizedmodel` actually relies on this C procedure.

4 Model fit conditional on the measure of effort $V_j^{(1)}$

4.1 Selection of the penalty parameter ν by cross-validation

4.1.1 Four possible criteria to measure the prediction error

In this section, we consider the measure of effort $V_j^{(1)}$. We first have used the function `crossvalidation` to identify the most suitable value of the penalty parameter ν.

Although we only present one criterion to measure the prediction error in the paper, we have considered four possible criteria to select the best value of the penalty parameter ν in our calculations. We presented in our paper the only theoretically funded and stable measure of the prediction error. However, we describe the other criteria that we used in this appendix. In all cases, the criterion was calculated with:

$$Q_g(\nu) = \sum_{r=1}^{R} Q_g^{(r)}(\nu)$$

with $g = 1, ..., 4$ defining the four possible criteria:

- $g = 1$: this criterion corresponds to the predicted log-likelihood of the validation dataset (this criterion is the one presented in the paper), i.e.:

$$Q_1^{(r)}(\nu) = \sum_{i=1}^{I}\sum_{j=1}^{J}\sum_{k=1}^{K} N_{ijk}^{(r)} \left\{ s_j + a_{ij}^{(-r)} + e_{jk}^{(-r)} + p_{ik}^{(-r)} - \log(R-1) \right\} - \exp\left\{ s_j + a_{ij}^{(-r)} + e_{jk}^{(r)} + p_{ik}^{(-r)} - \log(R-1) \right\}$$

  Again, we use here the same notations as in the paper.

- $g = 2$: this criterion is the sum of squared differences between predictions and observations:

$$Q_1^{(r)}(\nu) = \sum_{i=1}^{I}\sum_{j=1}^{J}\sum_{k=1}^{K} \left(N_{ijk}^{(r)} - \exp\left\{ s_j + a_{ij}^{(-r)} + e_{jk}^{(r)} + p_{ik}^{(-r)} - \log(R-1) \right\} \right)^2$$

- $g = 3$: the chi-squared statistics:

$$Q_1^{(r)}(\nu) = \sum_{i=1}^{I}\sum_{j=1}^{J}\sum_{k=1}^{K} \frac{\left(N_{ijk}^{(r)} - \exp\left\{ s_j + a_{ij}^{(-r)} + e_{jk}^{(r)} + p_{ik}^{(-r)} - \log(R-1) \right\} \right)^2}{\exp\left\{ s_j + a_{ij}^{(-r)} + e_{jk}^{(r)} + p_{ik}^{(-r)} - \log(R-1) \right\}}$$

- $g = 4$: the sum of the squared differences between observations and predictions divided by the predictions +1:

$$Q_1^{(r)}(\nu) = \sum_{i=1}^{I}\sum_{j=1}^{J}\sum_{k=1}^{K} \frac{\left(N_{ijk}^{(r)} - \exp\left\{ s_j + a_{ij}^{(-r)} + e_{jk}^{(r)} + p_{ik}^{(-r)} - \log(R-1) \right\} \right)^2}{\exp\left\{ s_j + a_{ij}^{(-r)} + e_{jk}^{(r)} + p_{ik}^{(-r)} - \log(R-1) \right\} + 1}$$

As noted above, the implementation of the cross-validation revealed that the criterion Q_1 was the best measure of the prediction error, so that we only presented the results for this criterion for the sake of concision.

4.1.2 Implementation of the cross-validation approach

We implemented the cross validation approach below (Warning! these calculations are very slow!!! it took 4 hours on a PC with a processor Intel© core I5):

```
vv <- sapply(1:length(SAR@data$codeSAR), function(i) {
    mean(Vj1[as.character(dfdata$SAR) == as.character(SAR@data$codeSAR)[i]])
})
vv2 <- sapply(1:length(SAR@data$codeSAR), function(i) {
    mean(dfdata$Sj[as.character(dfdata$SAR) == as.character(SAR@data$codeSAR)[i]])
})
dfVj <- data.frame(nom = factor(SAR@data$codeSAR),
    Vj = vv, Sj = vv2)

## Then we make sure that the variable SAR in the
## data.frame scsldetect has the exact same set of
## levels as the first column of dfVj
scsldetect$SAR <- factor(scsldetect$SAR, levels = levels(dfVj[,
    1]))
## And we make sure that the other vectors are
## factors
scsldetect$Species <- factor(scsldetect$Species)
scsldetect$Status <- factor(scsldetect$Status)
scsldetect$Year <- factor(scsldetect$Year)

cvpra <- crossvalidation(scsldetect$Species, scsldetect$SAR,
    scsldetect$Status, scsldetect$Year, dfVj, vecnu = c(seq(0,
        0.5, by = 0.1), seq(0.6, 2, by = 0.2)), proximities = prx,
    control = list(verbose = TRUE, stopCrit = 1e-07))
```

The object `cvpra` contains all the information required for the calculation of the four criteria, as well as their decomposition per species. The function `global.crossvalCisstat` is used to derive the global criteria $Q_g(\nu)$ from this information:

```
glo <- global.crossvalCisstat(cvpra)
```

We can plot these criteria for the different values of ν:

```
plot(glo, xlim = c(0, 0.8), withgroups = FALSE)
```

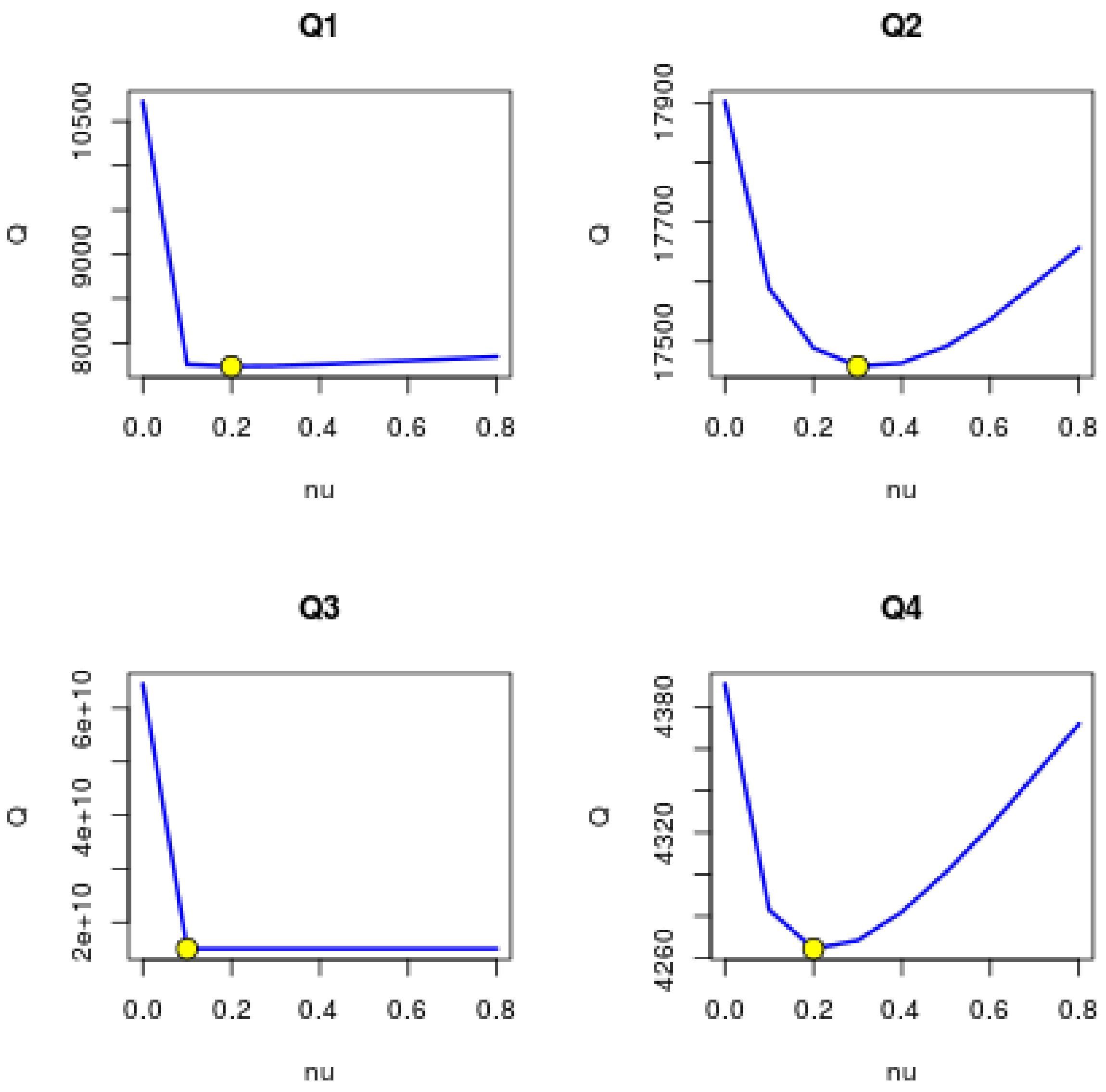

All these criteria reach a minimum around 0.2–0.4. Note that the criterion Q_3 is not a very good measure of the prediction error: the denominator of this criterion gives a too large weight to small values. For example, when the number of detected animals for a given combination of species, spatial unit and status is equal to 1, and when the predicted value is equal to 10^{-4}, then, this observation contributes to $\approx$ 10000 to this criterion, which is absurd given the small difference between these two values. This explains why this criterion takes such large values. We will not consider this criterion in the rest of this document.

We have also examined the contribution of each species to each criterion. These contributions are plotted below for the criterion Q_1:

```
plot(cvpra, xlim = c(0.1, 0.8), withgroups = FALSE)
```

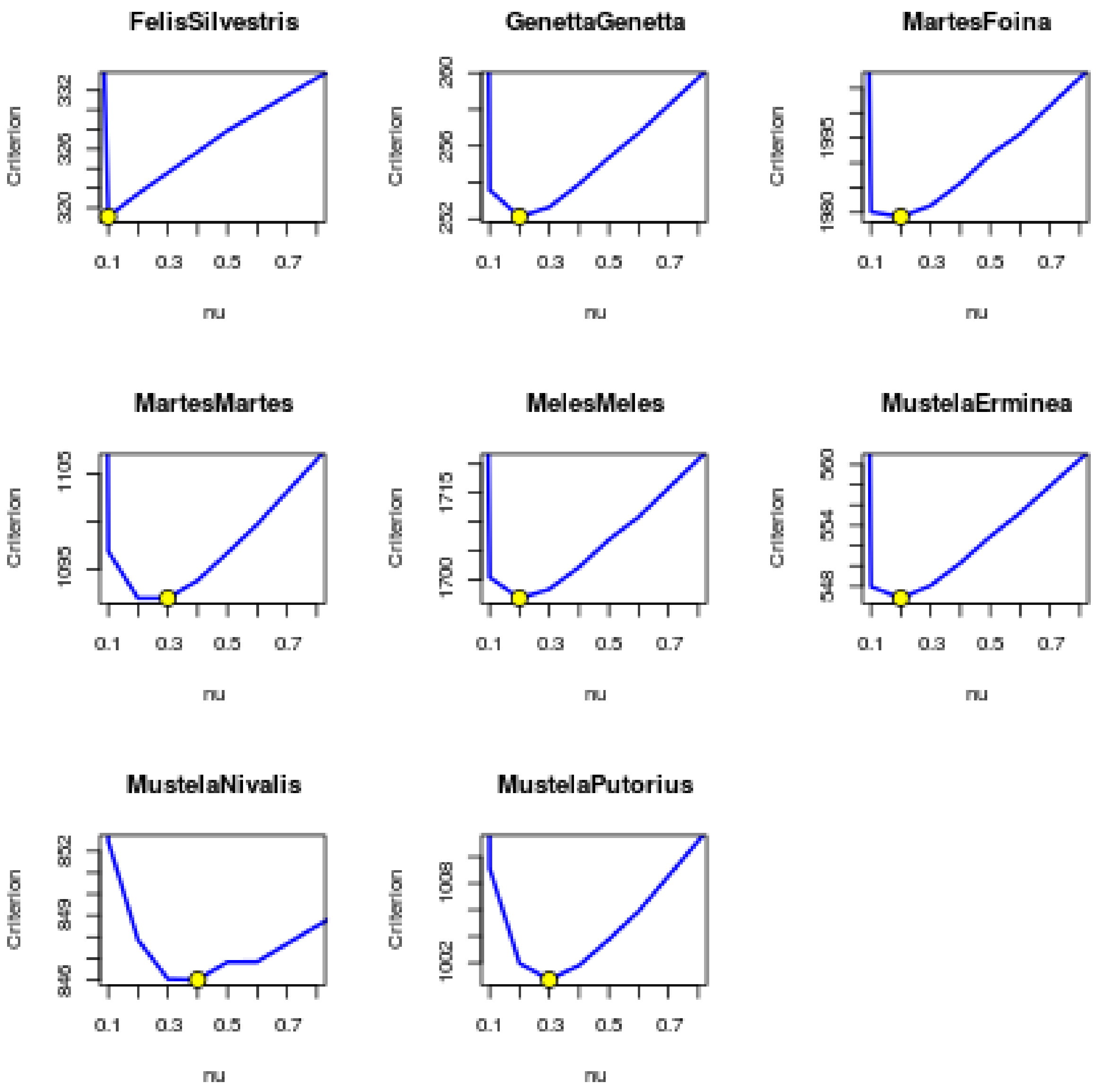

The value of the criterion is small for all species for $\nu = 0.3$. The results for the criterion Q_2 are plotted below:

```
plot(cvpra, criterion = 2, xlim = c(0, 0.8), withgroups = FALSE)
```

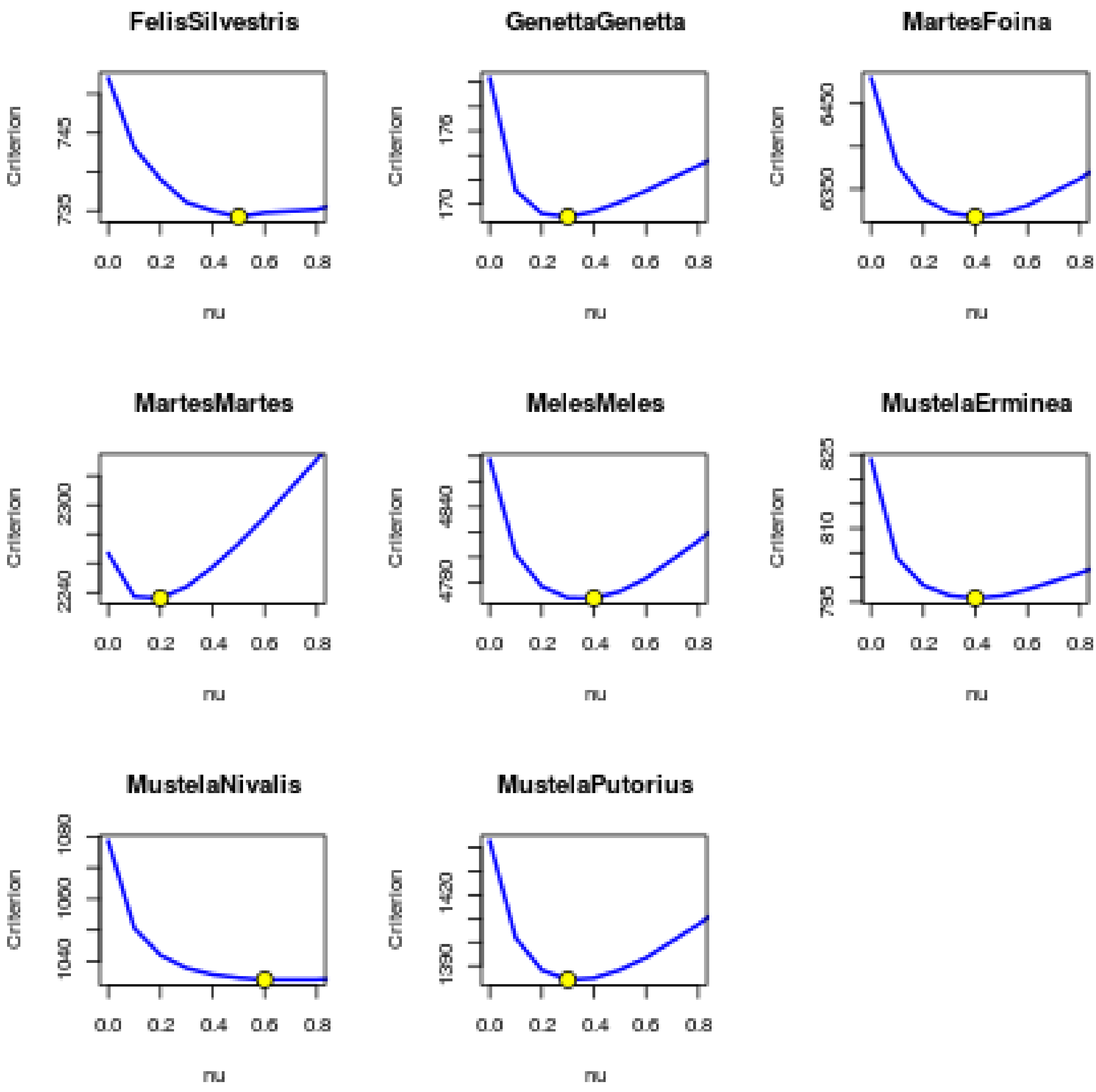

The smallest values of the criteron are more variable than for Q_1 (ranging from 0.2 to 0.6). A value of the penalty equal to 0.3 corresponds to a small value of this criterion for all species.

Note that the spatial units with a large number of detected animals have a too large weight in the calculation of this criterion. Indeed, a difference between the observed and the predicted number of detections equal to 10 should not have the same weight if the actual number of detections is equal to 0 or to 1000. For this reason, we proposed the use of the criterion Q_4, which standardizes these differences in an *ad hoc* way:

```
plot(cvpra, criterion = 4, xlim = c(0, 0.8), withgroups = FALSE)
```

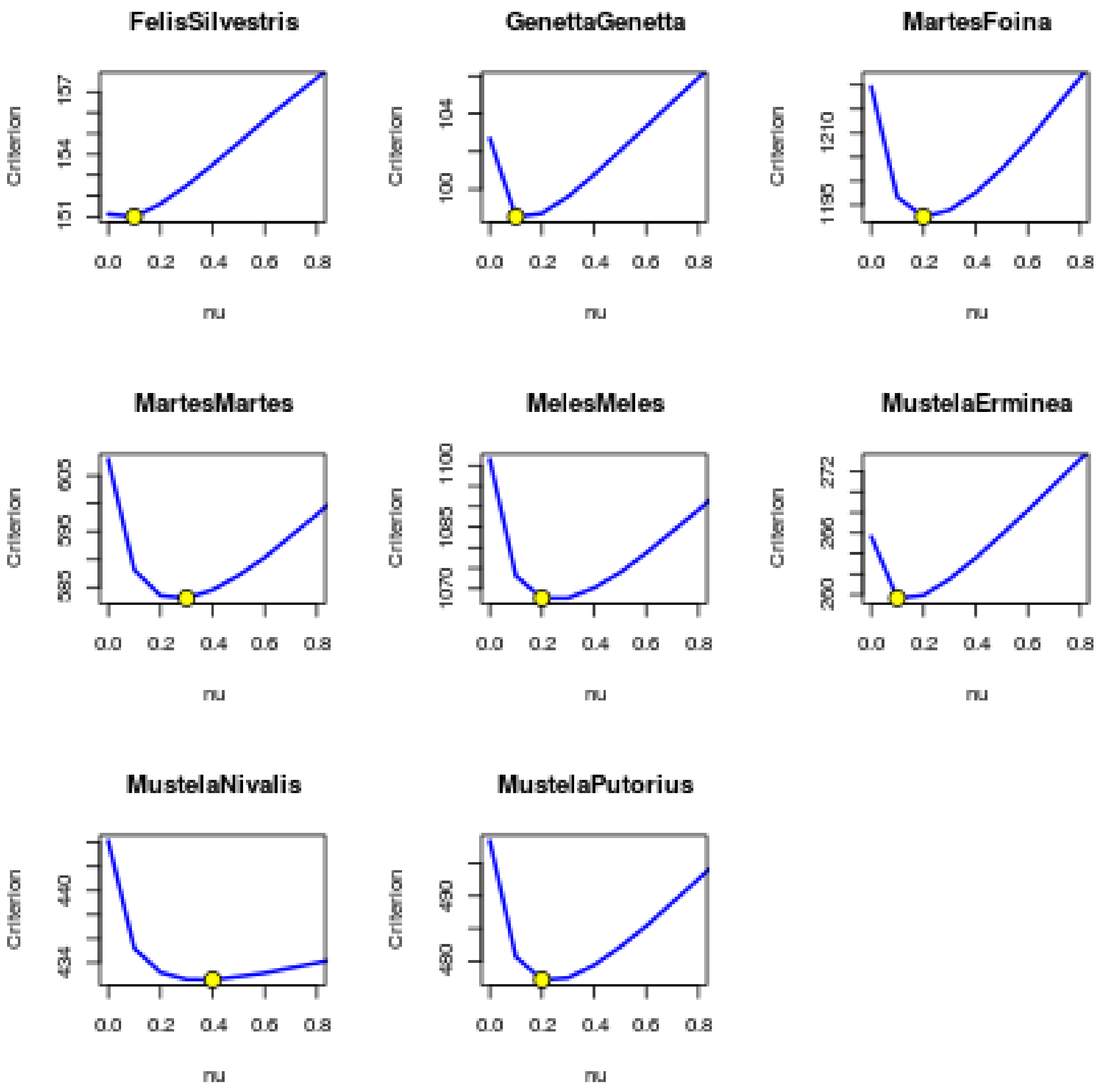

For this criterion, we obtain results pretty similar to those obtained for the criterion Q_1. For most species, the value $\nu = 0.2$ is optimal.

Given the previous results, the prediction error seems to be reasonably small for all species and all criteria with $\nu = 0.3$. This is the value that we have chosen to fit our model.

4.2 Model fit with $\nu = 0.3$

We fitted the model with a penalty parameter $\nu = 0.3$ below:

```
dfdata$Sp <- factor(dfdata$Sp)
dfdata$SAR <- factor(dfdata$SAR)
dfdata$Sta <- factor(dfdata$Sta)

modVj1 <- penalizedmodel(dfdata$Sp, dfdata$SAR, dfdata$Sta,
    dfdata$Nijk, Vj1, Area = dfdata$Sj, proximities = prx,
    nu = 0.3, control = list(typealgo = 3, verbose = TRUE,
        stopCrit = 1e-08))
```

We can map the estimated relative densities for each species:

```
## The library Hmisc is required to use the function
## cut2 below (this function cuts a numeric variable
## into n groups of equal size)
library(Hmisc)

## This function scales a numeric variable between 0
## and 1 for further mapping with the function
## grey()
togrey <- function(x) {
    0.8 * (x - min(x))/diff(range(x)) + 0.1
}


par(mfrow = c(3, 3), mar = c(0, 0, 2, 0))
tmp <- sapply(1:8, function(i) {
    plot(SAR, col = grey(togrey(-as.numeric(cut2(modVj1$aij[,
        i], g = 20)))), border = NA)
    title(levels(dfdata$Sp)[i])
})
```

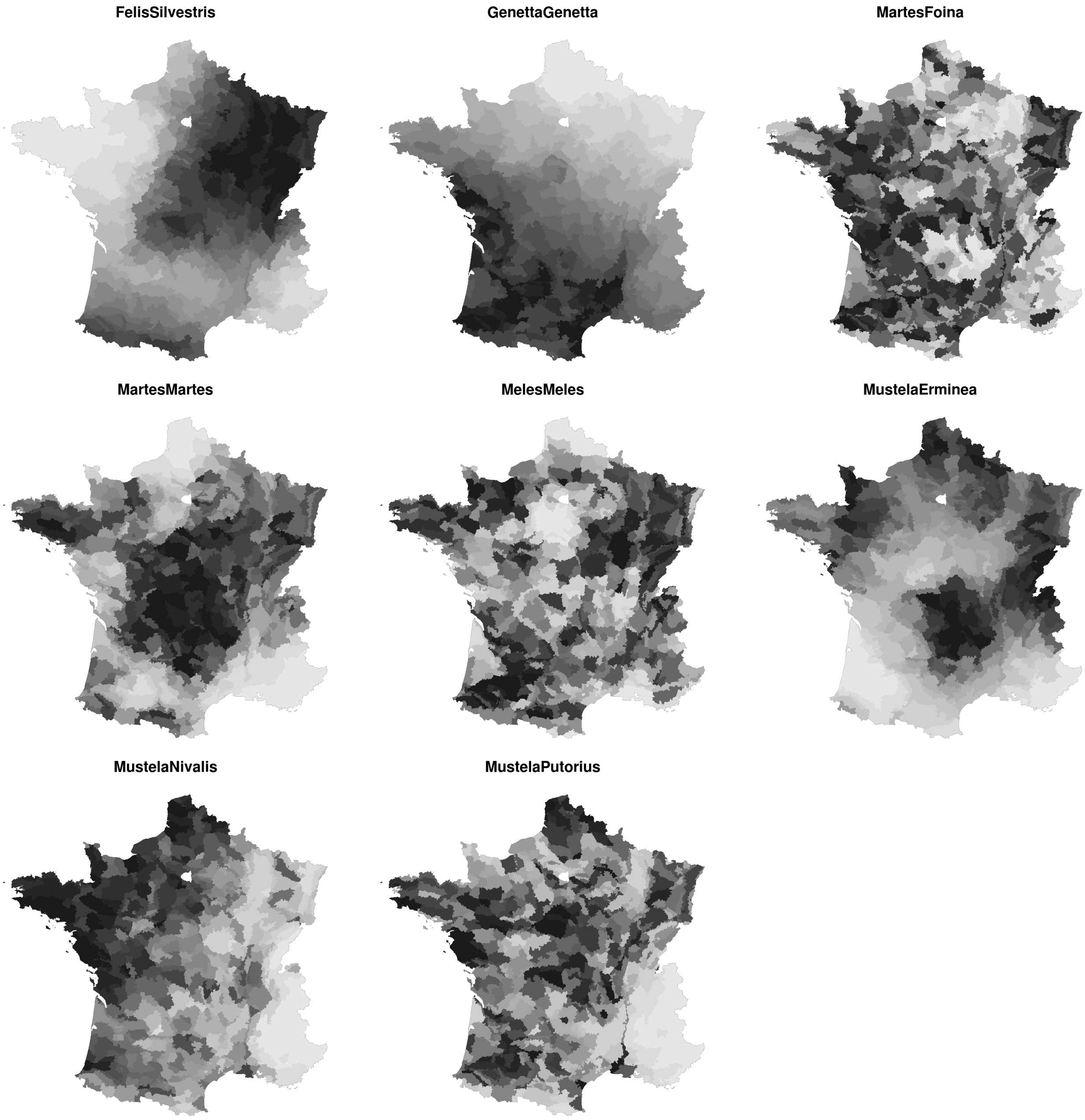

We can also map the sampling effort known for the dead animals and estimate for the living animals:

```
par(mfrow = c(2, 1), mar = c(0, 0, 2, 0))
plot(SAR, col = grey(togrey(-modVj1$ejk[, 1])), border = NA)
title("Sampling effort; dead animals")
box()
## To improve the visualization, replace the zero
## estimated log-effort (theoretically equal to
## -infinity, but arbitrarily set to -30 by our
## function) by the smallest non-null estimated
## effort (2.7):
el <- modVj1$ejk[, 2]
el[el < 0] <- min(el[el >= 0])
plot(SAR, col = grey(togrey(-el)), border = NA)
title("Sampling effort; living animals")
box()
```

Sampling effort; dead animals

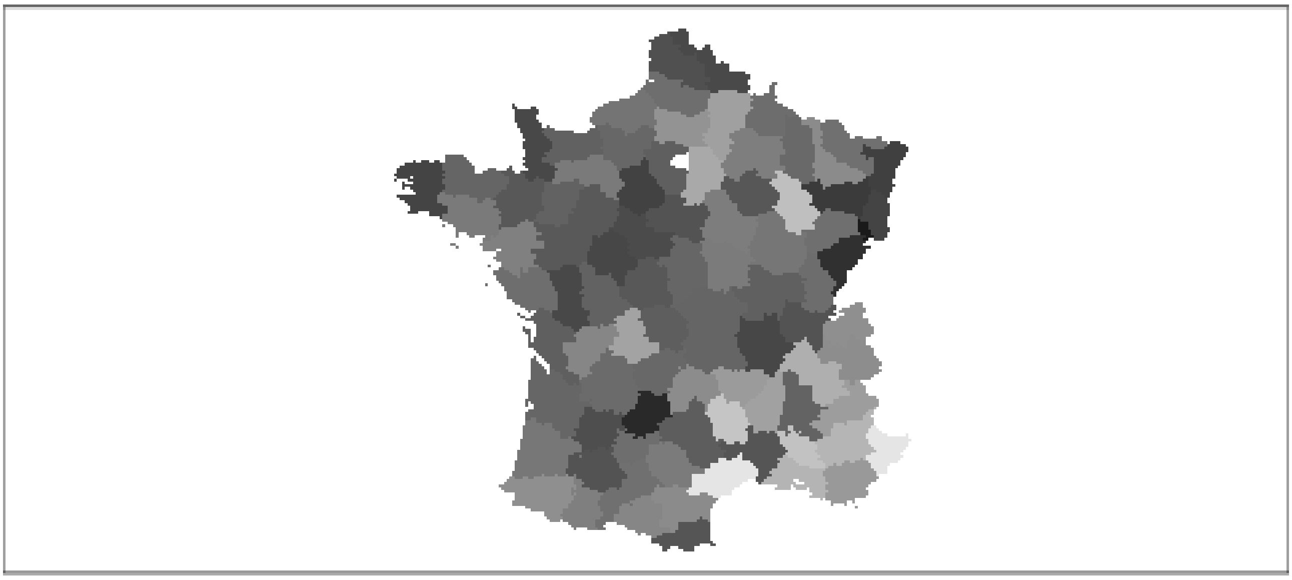

Sampling effort; living animals

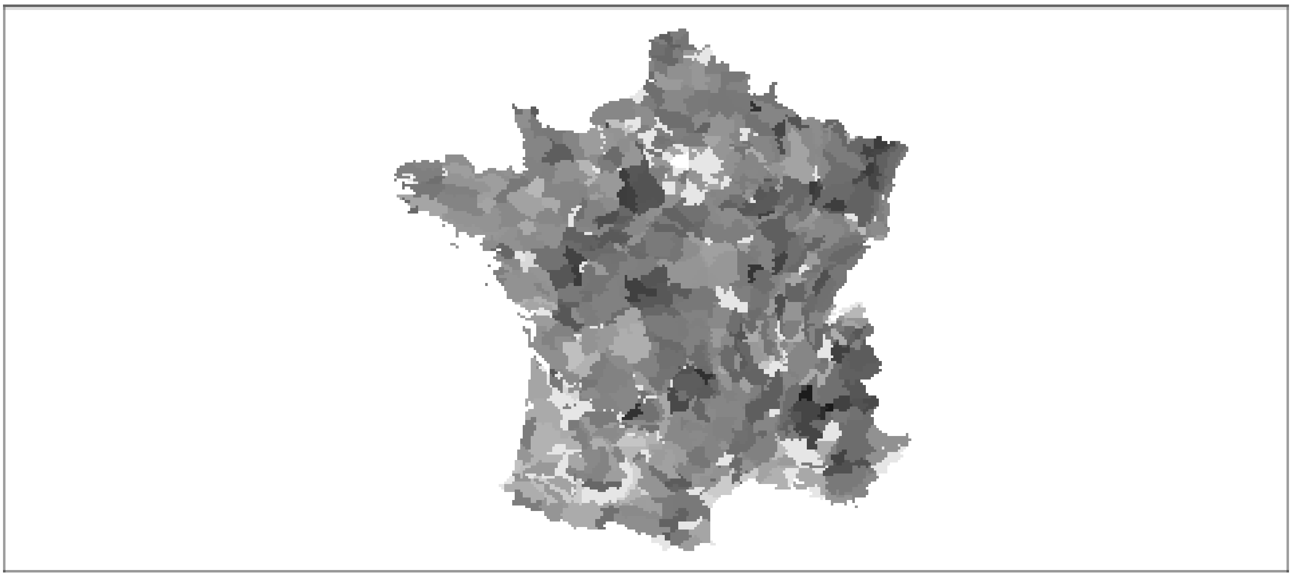

5 Model fit conditional on the measure of effort $V_j^{(2)}$

5.1 Selection of the penalty parameter ν by cross-validation

We have fitted our model conditional on the measure of effort $V_j^{(2)}$, i.e. under the more complicated model of the distribution of effort within a department. We used an approach identical to the one described in the previous section:

```
## We recalculate the dataframe dfVj with this
## alternative measure of effort
dfVj <- data.frame(nom = factor(SAR@data$codeSAR),
    Vj = Vj2, Sj = vv2)
## Then we make sure that the variable SAR in the
```

```
## data.frame scsldetect has the exact same set of
## levels as the first column of dfVj
scsldetect$SAR <- factor(scsldetect$SAR, levels = levels(dfVj[,
    1]))
## And we make sure that the other vectors are
## factors
scsldetect$Species <- factor(scsldetect$Species)
scsldetect$Status <- factor(scsldetect$Status)
scsldetect$Year <- factor(scsldetect$Year)


cvpra2 <- crossvalidation(scsldetect$Species, scsldetect$SAR,
    scsldetect$Status, scsldetect$Year, dfVj, vecnu = c(seq(0,
        0.5, by = 0.1), seq(0.6, 2, by = 0.2)), proximities = prx)
fooo <- function(x1, x2) c(x2, x1[-c(1, 2, 3)])

cvpra2[[2]] <- fooo(cvpra2[[2]], cvprafin2[[2]])
for (i in 1:4) {
    for (j in 1:8) {
        cvpra2[[1]][[i]][[j]] <- rbind(cvprafin2[[1]][[i]][[j]],
            cvpra2[[1]][[i]][[j]][-c(1, 2, 3), ])
    }
}
```

The object `cvpra2` contains all the information required for the calculation of the four criteria, as well as their decomposition per species. The function `global.crossvalCisstat` is used to derive the global criteria $Q_g(\nu)$ from this information:

```
glo2 <- global.crossvalCisstat(cvpra2)
```

We can plot these criteria for the different values of ν:

```
plot(glo2)
```

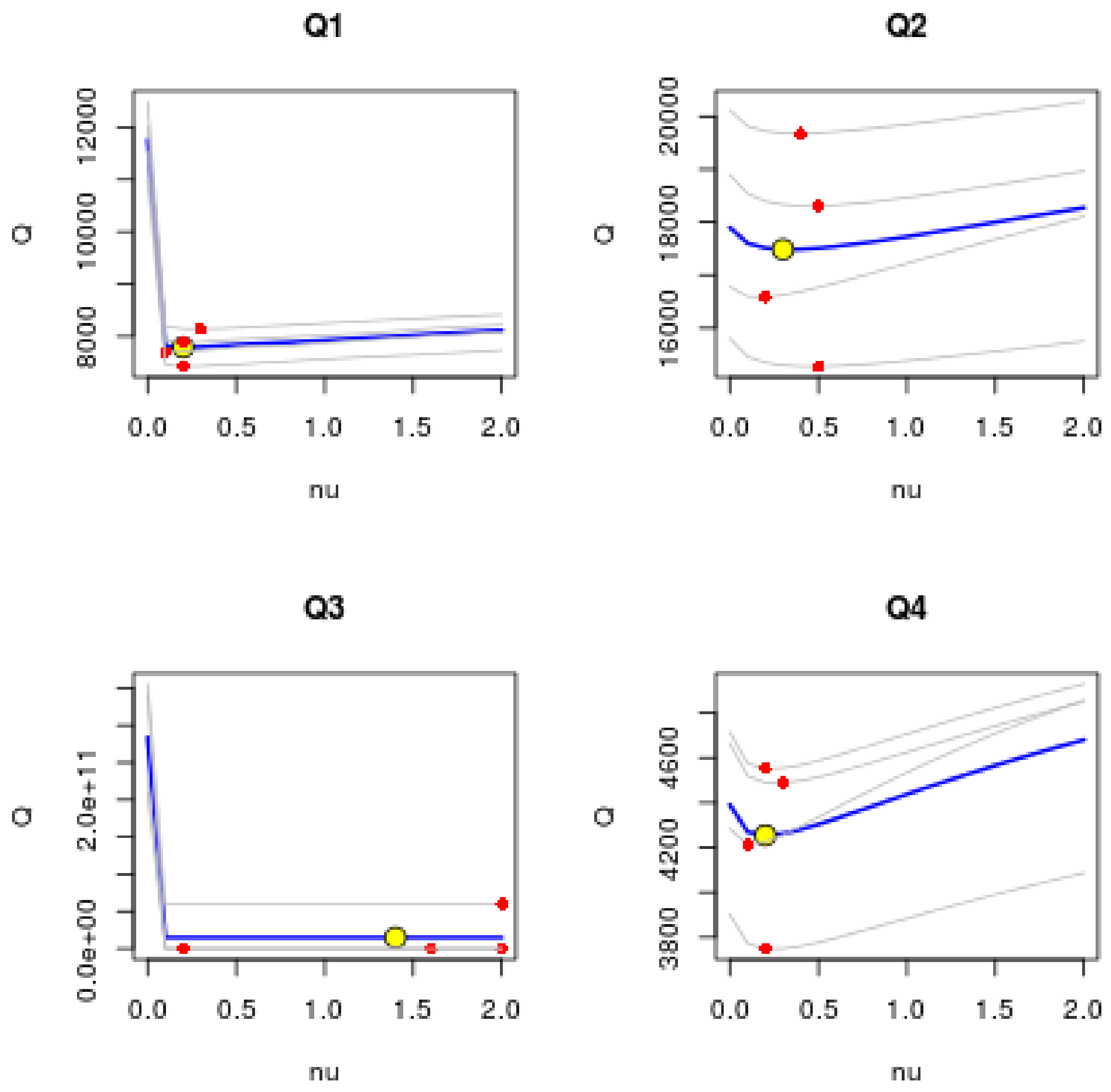

All the criteria indicate that ν=0.2 – 0.3 is a good choice for the regularization. The examination of these results for each species confirmed this hypothesis. In particular, for the criterion Q_1:

```
plot(cvpra2, withgroups = F, xlim = c(0.1, 1))
```

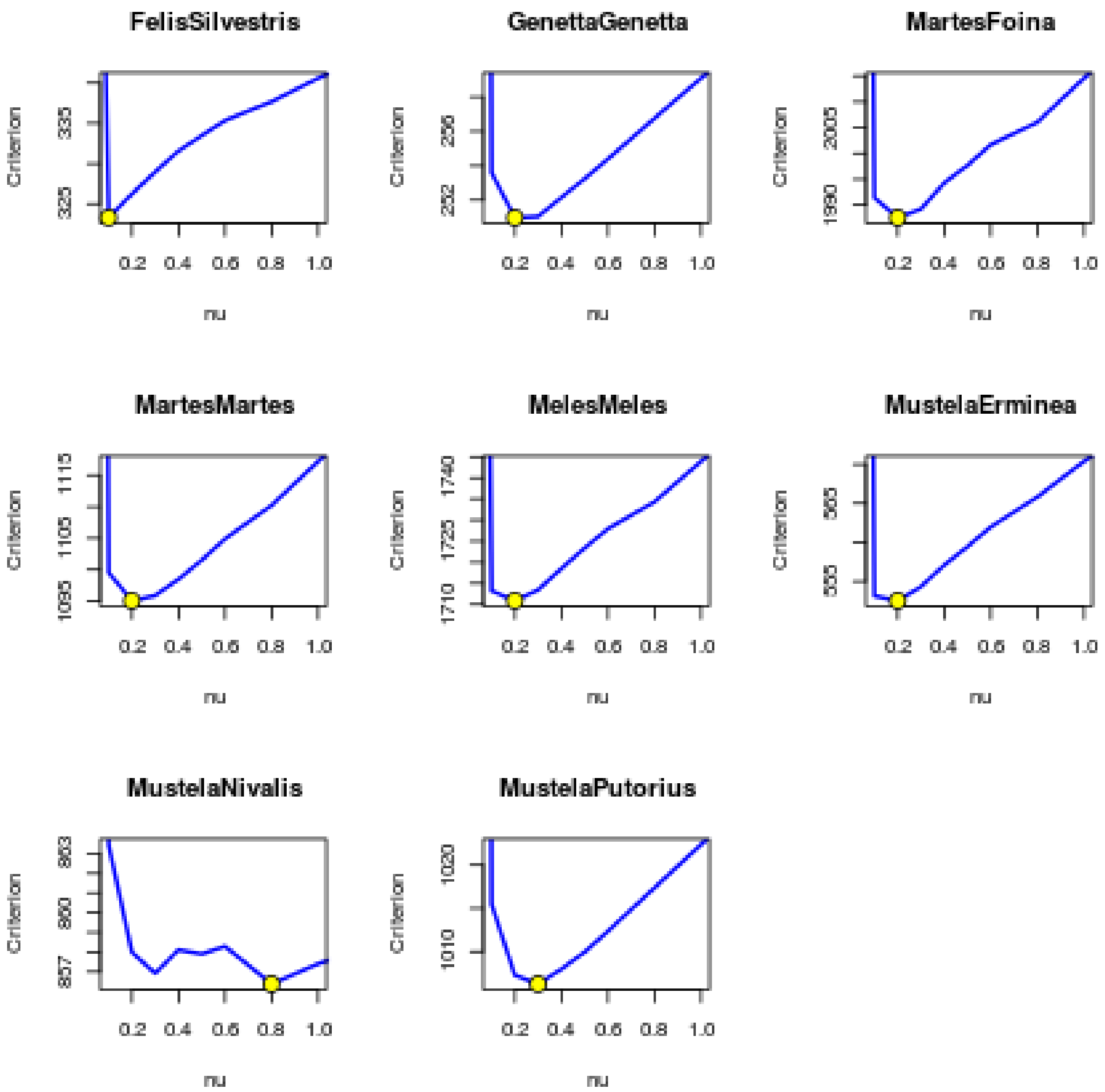

For the weasel, this criterion suggests that a stronger regularization is required. On the other hand, the criterion Q_4 suggests that a penalty roughly equal to 0.2–0.3 is a good choice for this species:

```
plot(cvpra2, withgroups = F, xlim = c(0.1, 1), criterion = 4)
```

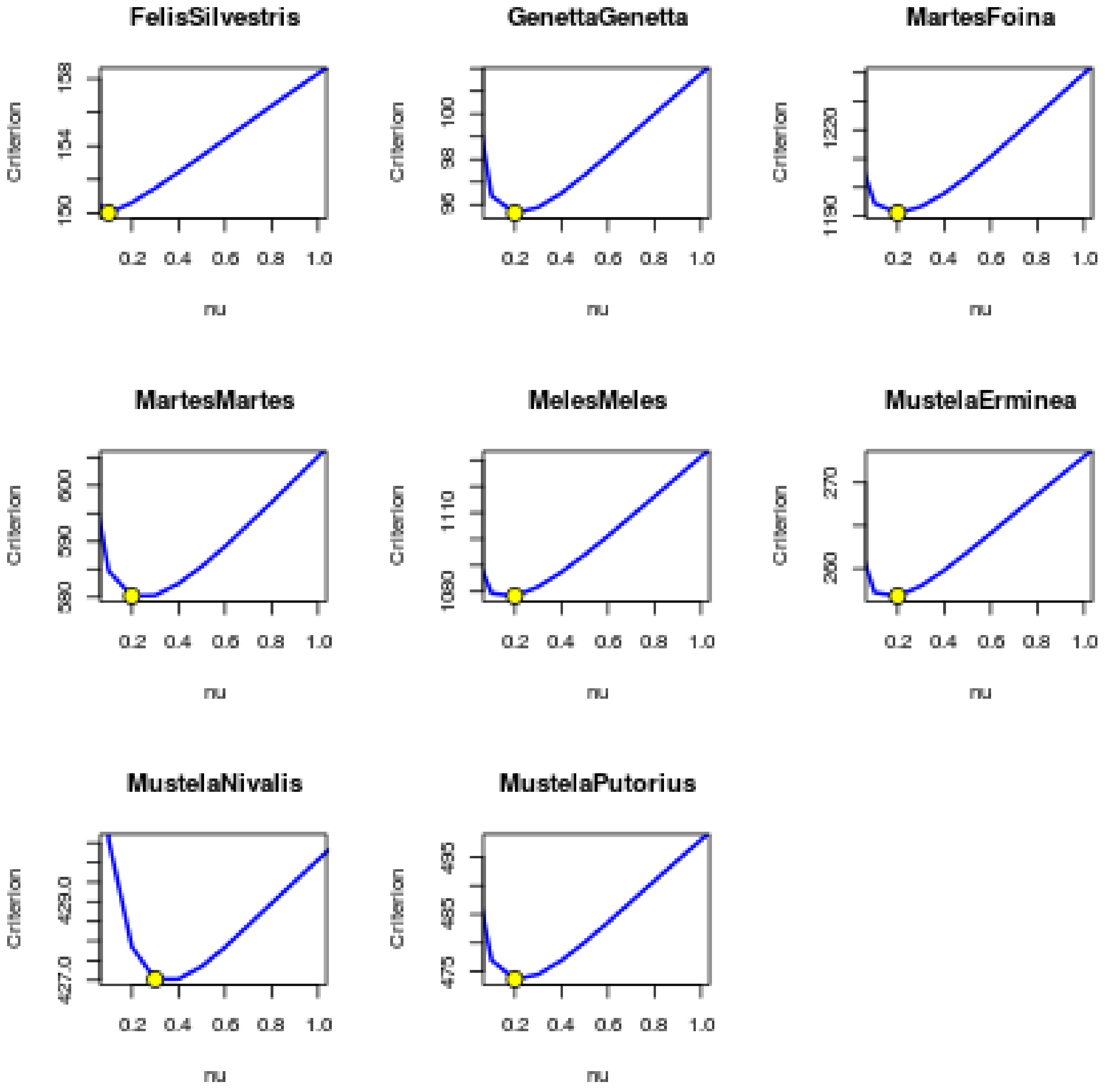

For the sake of simplicity, we have considered the penalty parameter $\nu = 0.3$ for the model based on this measure of effort, which corresponds to small prediction error for all species.

5.2 Model fit with $\nu = 0.3$

We fitted the model with a penalty parameter $\nu = 0.3$ below:

```
Vjab <- sapply(as.character(dfdata$SAR), function(x) {
    Vj2[as.character(SAR@data$codeSAR) == x]
})

modVj2 <- penalizedmodel(factor(dfdata$Sp), factor(dfdata$SAR),
    factor(dfdata$Sta), dfdata$Nijk, Vjab, Area = dfdata$Sj,
    proximities = prx, nu = 0.3, control = list(typealgo = 3,
        verbose = TRUE, stopCrit = 1e-08))
```

We can map the estimated relative densities for each species:

```
par(mfrow = c(3, 3), mar = c(0, 0, 2, 0))
tmp <- sapply(1:8, function(i) {
    plot(SAR, col = grey(togrey(-as.numeric(cut2(modVj2$aij[,
        i], g = 20)))), border = NA)
    title(levels(scsldetect$Species)[i])
})
```

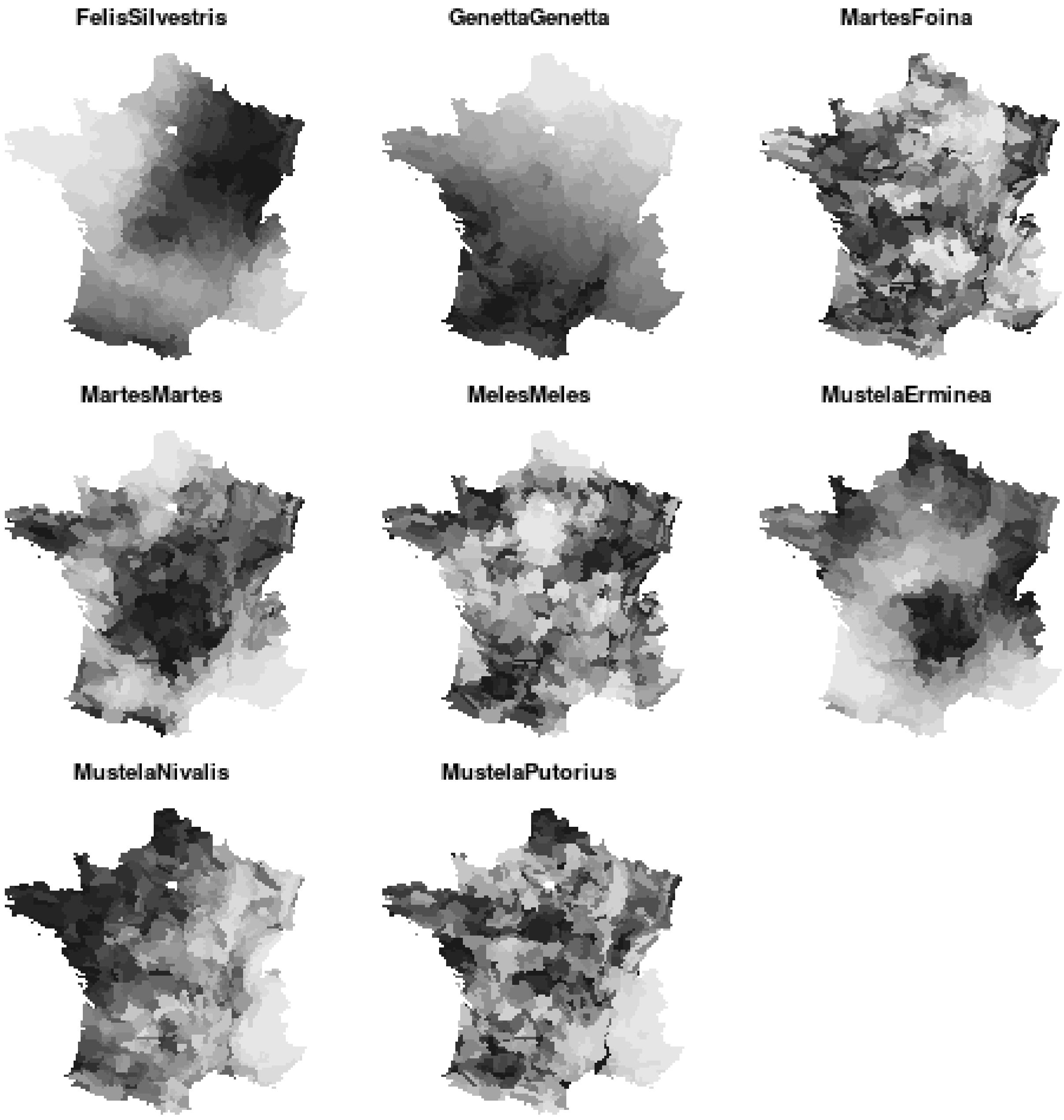

6 Which effort measure is the best?

6.1 Implementation of a cross-validation approach

To identify the most suitable measure of the sampling effort for the dead, we have implemented a cross-validation approach. We have considered the even years (2002, 2004) and the odd years (2003, 2005) separately. We have fitted a model using the data collected during the even years and we have measured the prediction error using our four criteria on the odd years, and conversely.

We first formatted the data for the implementation of this crossvalidation approach, considering first the measure $V_j^{(1)}$:

```
## We first prepare the data
scsldetect$Year <- as.numeric(as.character(scsldetect$Year))
scsl <- scsldetect
effortpercar <- effortpercar
linumsar <- linumsar
cars <- carsscsl

#### 1. Sampling effort of the dead supposed to be
#### constant within a department

## model 1
g <- 1
obsa <- scsl[scsl$Year%%2 == (g - 1), ]
obsb <- scsl[scsl$Year%%2 != (g - 1), ]
veha <- cars[cars$Year%%2 == (g - 1), ]
vehb <- cars[cars$Year%%2 != (g - 1), ]

## calculation of the effort for odd and even years
## (Vja and Vjb)
Vja <- sapply(as.character(SAR@data$codeSAR), function(y) {
    ## the department code
    x <- substr(y, 1, 2)
    ## remember that the departments 91, 95 and 78 are
    ## coded 78:
    if (any(c("91", "95") == x))
        x <- "78"
    ## Calculation of the total effort in the
    ## department, standardized by the area
    a <- veha$Car[as.character(veha$Department) ==
        x]
    b <- veha$Km[as.character(veha$Department) == x]
    if (all(is.na(b))) {
        b <- carsscsl$Km[as.character(carsscsl$Department) ==
            x]
    }
    c <- department@data$Area[department@data$Code ==
        x]
    length(a) * mean(na.omit(b))/c
})
Vjb <- sapply(as.character(SAR@data$codeSAR), function(y) {
    ## the department code
    x <- substr(y, 1, 2)
    ## remember that the departments 91, 95 and 78 are
    ## coded 78:
```

```
    if (any(c("91", "95") == x))
        x <- "78"
    ## Calculation of the total effort in the
    ## department, standardized by the area
    a <- vehb$Car[as.character(vehb$Department) ==
        x]
    b <- vehb$Km[as.character(vehb$Department) == x]
    if (all(is.na(b))) {
        b <- carsscsl$Km[as.character(carsscsl$Department) ==
            x]
    }
    c <- department@data$Area[department@data$Code ==
        x]
    length(a) * mean(na.omit(b))/c

})
Vjab <- sapply(as.character(dfdata$SAR), function(x) Vja[names(Vja) ==
    x])
Vjbb <- sapply(as.character(dfdata$SAR), function(x) Vjb[names(Vjb) ==
    x])

## construction of the calibration and validation
## datasets
dfdatasara <- do.call("rbind", lapply(levels(factor(scsl$Species)),
    function(i) {
        ## For each species, we keep only the detected
        ## animals of the species
        obse <- obsa[obsa$Species == i, ]

        ## For the dead and living animals
        do.call("rbind", lapply(c("D", "L"), function(k) {
            ## and for each sar
            do.call("rbind", lapply(SAR@data$codeSAR,
                function(sara) {
                  ## return the number of detected animals belonging
                  ## to dataset a
                  data.frame(Nijk = nrow(obse[as.character(obse$SAR) ==
                    sara & obse$Status == k, ]), Sp = i,
                    Sta = k, SAR = sara)
                }))
        }))
    }))

dfdatasarb <- do.call("rbind", lapply(levels(factor(scsl$Species)),
    function(i) {
        ## For each species, we keep only the detected
        ## animals of the species
        obse <- obsb[obsb$Species == i, ]

        ## For the dead and living animals
        do.call("rbind", lapply(c("D", "L"), function(k) {
            ## and for each sar
            do.call("rbind", lapply(SAR@data$codeSAR,
                function(sara) {
                  ## return the number of detected animals belonging
                  ## to dataset b
                  data.frame(Nijk = nrow(obse[as.character(obse$SAR) ==
```

```
                     sara & obse$Status == k, ]), Sp = i,
                     Sta = k, SAR = sara)
                 }))
         }))
     }))


surf <- SAR@data$Area
surfb <- sapply(as.character(dfdata$SAR), function(x) {
    surf[as.character(SAR@data$codeSAR) == x]
})
dfdatasara <- data.frame(dfdatasara, Vj = Vjab, Area = surfb)
dfdatasarb <- data.frame(dfdatasarb, Vj = Vjbb, Area = surfb)
dfdatasara$Sp <- factor(dfdatasara$Sp)
dfdatasarb$Sp <- factor(dfdatasarb$Sp)
dfdatasara$SAR <- factor(dfdatasara$SAR)
dfdatasarb$SAR <- factor(dfdatasarb$SAR)
dfdatasara$Sta <- factor(dfdatasara$Sta)
dfdatasarb$Sta <- factor(dfdatasarb$Sta)
```

We then fitted the models for odd and even years respectively:

```
## Model fit
modpraa <- penalizedmodel(dfdatasara$Sp, dfdatasara$SAR,
    dfdatasara$Sta, dfdatasara$Nijk, dfdatasara$Vj,
    Area = dfdatasara$Area, proximities = prx, nu = 0.3,
    control = list(typealgo = 3, verbose = TRUE, stopCrit = 1e-07))
modprab <- penalizedmodel(dfdatasarb$Sp, dfdatasarb$SAR,
    dfdatasarb$Sta, dfdatasarb$Nijk, dfdatasarb$Vj,
    Area = dfdatasarb$Area, proximities = prx, nu = 0.3,
    control = list(typealgo = 3, verbose = TRUE, stopCrit = 1e-07))
```

We then calculated the different criteria measuring the prediction error:

```
## Prediction for the dead
preb <- dfdatasarb[dfdatasarb$Sta == "D", ]
prea <- dfdatasarb[dfdatasara$Sta == "D", ]
Yhatbfroma <- sapply(1:length(preb$Sp), function(i) {
    exp(modpraa$aij[preb$SAR[i], preb$Sp[i]] + log(preb$Vj[i]))
}) * preb$Area
Yhatafromb <- sapply(1:length(prea$Sp), function(i) {
    exp(modprab$aij[prea$SAR[i], prea$Sp[i]] + log(prea$Vj[i]))
}) * prea$Area


## Prediction error according to the four criteria
Q1 <- sum(-dpois(preb$Nijk, Yhatbfroma, log = TRUE))
Q2 <- sum((Yhatbfroma - preb$Nijk)^2)
Q3 <- sum(((Yhatbfroma - preb$Nijk)^2)/Yhatbfroma)
Q4 <- sum(((Yhatbfroma - preb$Nijk)^2)/(Yhatbfroma +
    1))
critbfroma <- c(Q1, Q2, Q3, Q4)

Q1 <- sum(-dpois(prea$Nijk, Yhatafromb, log = TRUE))
Q2 <- sum((Yhatafromb - prea$Nijk)^2)
Q3 <- sum(((Yhatafromb - prea$Nijk)^2)/Yhatafromb)
Q4 <- sum(((Yhatafromb - prea$Nijk)^2)/(Yhatafromb +
```

```
    1))
critafromb <- c(Q1, Q2, Q3, Q4)
aa <- rbind(critafromb, critbfroma)
aa <- as.data.frame(rbind(aa, apply(aa, 2, mean)))
names(aa) <- c("Q1", "Q2", "Q3", "Q4")
row.names(aa) <- c("EvenFromOdd", "OddFromEven", "Mean")
res1 <- (prea$Nijk - Yhatafromb)/sqrt(Yhatafromb)
res2 <- (preb$Nijk - Yhatbfroma)/sqrt(Yhatbfroma)
```

We also prepared the data for the implementation of the cross-validation approach conditional on the measure $V_j^{(2)}$.

```
#### 2. Alternative measure of effort
Vjb <- .Call("Calculeffort", effortpercar, linumsar,
    as.integer(cars$Year%%2), as.double(SAR@data$Area),
    as.integer(nrow(SAR)), PACKAGE = "scsl")
Vja <- .Call("Calculeffort", effortpercar, linumsar,
    as.integer((cars$Year%%2) == 0), as.double(SAR@data$Area),
    as.integer(nrow(SAR)), PACKAGE = "scsl")
Vjab <- sapply(as.character(dfdata$SAR), function(x) {
    Vja[as.character(SAR@data$codeSAR) == x]
})
Vjbb <- sapply(as.character(dfdata$SAR), function(x) {
    Vjb[as.character(SAR@data$codeSAR) == x]
})
dfdatasara$Vj <- Vjab
dfdatasarb$Vj <- Vjbb
```

We then fitted the models for odd and even years respectively, conditional on this alternative measure of effort:

```
modpraa <- penalizedmodel(dfdatasara$Sp, dfdatasara$SAR,
    dfdatasara$Sta, dfdatasara$Nijk, dfdatasara$Vj,
    Area = dfdatasara$Area, proximities = prx, nu = 0.3,
    control = list(typealgo = 3, verbose = TRUE, stopCrit = 1e-07))
modprab <- penalizedmodel(dfdatasarb$Sp, dfdatasarb$SAR,
    dfdatasarb$Sta, dfdatasarb$Nijk, dfdatasarb$Vj,
    Area = dfdatasarb$Area, proximities = prx, nu = 0.3,
    control = list(typealgo = 3, verbose = TRUE, stopCrit = 1e-07))
```

We then calculated the different criteria measuring the prediction error:

```
## Prediction for the dead
preb <- dfdatasarb[dfdatasarb$Sta == "D", ]
prea <- dfdatasarb[dfdatasara$Sta == "D", ]
Yhatbfroma <- sapply(1:length(preb$Sp), function(i) {
    exp(modpraa$aij[preb$SAR[i], preb$Sp[i]] + log(preb$Vj[i]))
}) * preb$Area
Yhatafromb <- sapply(1:length(prea$Sp), function(i) {
    exp(modprab$aij[prea$SAR[i], prea$Sp[i]] + log(prea$Vj[i]))
}) * prea$Area

## Prediction error according to the four criteria
Q1 <- sum(-dpois(preb$Nijk, Yhatbfroma, log = TRUE))
Q2 <- sum((Yhatbfroma - preb$Nijk)^2)
Q3 <- sum(((Yhatbfroma - preb$Nijk)^2)/Yhatbfroma)
Q4 <- sum(((Yhatbfroma - preb$Nijk)^2)/(Yhatbfroma +
    1))
```

```
critbfroma <- c(Q1, Q2, Q3, Q4)

Q1 <- sum(-dpois(prea$Nijk, Yhatafromb, log = TRUE))
Q2 <- sum((Yhatafromb - prea$Nijk)^2)
Q3 <- sum(((Yhatafromb - prea$Nijk)^2)/Yhatafromb)
Q4 <- sum(((Yhatafromb - prea$Nijk)^2)/(Yhatafromb +
    1))
critafromb <- c(Q1, Q2, Q3, Q4)
aa2 <- rbind(critafromb, critbfroma)
aa2 <- as.data.frame(rbind(aa2, apply(aa2, 2, mean)))
names(aa2) <- c("Q1", "Q2", "Q3", "Q4")
row.names(aa2) <- c("EvenFromOdd", "OddFromEven", "mean")
res1b <- (prea$Nijk - Yhatafromb)/sqrt(Yhatafromb)
res2b <- (preb$Nijk - Yhatbfroma)/sqrt(Yhatbfroma)
```

6.2 Results

We present the value of the criteria for the measure $V_j^{(1)}$ below:

```
aa

##                Q1    Q2   Q3   Q4
## EvenFromOdd 4388  5979 2588 1104
## OddFromEven 6092 31266 8063 4060
## Mean        5240 18623 5326 2582
```

We present the value of the criteria for the measure $V_j^{(2)}$ below:

```
aa2

##                Q1    Q2    Q3   Q4
## EvenFromOdd 4384  6124  4511 1244
## OddFromEven 6551 47556 13957 4941
## mean        5468 26840  9234 3092
```

It is clear the measure $V_j^{(1)}$ was better according to all criteria. A quantile-quantile plot displaying the standardized deviations between predictions and observations (i.e. the residuals) for the two measures of effort indicates that the residuals are much greater for the measure $V_j^{(2)}$:

```
par(mfrow = c(2, 1))
qqplot(res1, res1b, xlab = "Residuals constant effort",
    ylab = "Residuals alternative effort", main = "Prediction even years",
    cex = 1.5)
abline(0, 1, col = "red", lwd = 2)
qqplot(res2, res2b, xlab = "Residuals constant effort",
    ylab = "Residuals alternative effort", main = "Prediction odd years",
    cex = 1.5)
abline(0, 1, col = "red", lwd = 2)
```

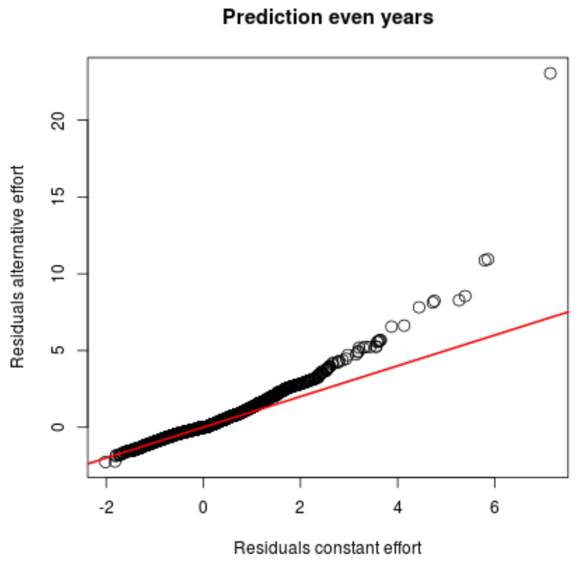

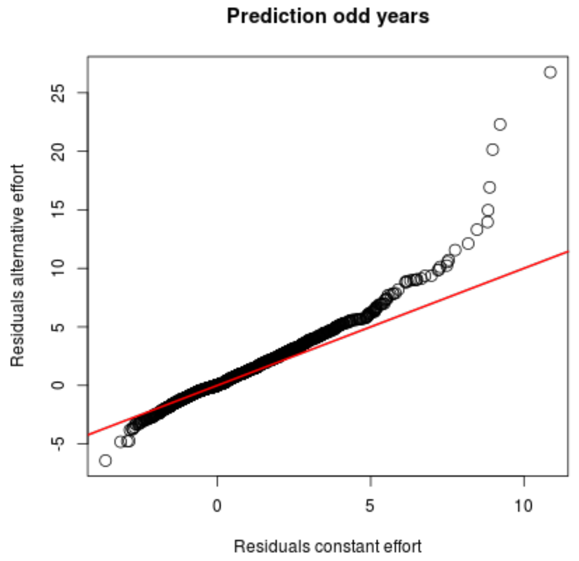

We therefore considered the measure $V_j^{(1)}$ because it was characterized by both smaller prediction errors and smaller prediction residuals than the alternative measure.

7 Is a purely spatial regularization a better choice to fit our model?

7.1 Implementation of a cross-validation approach

As noted in the paper, we maximize a *regularized* log-likelihood to account for a spatial and environmental autocorrelation. In this section, we compared this approach to an approach where a *purely spatial regularization* is implemented. More precisely, we tried to maximize the following quantity:

$$\log \mathcal{L} - \sum_{i=1}^{I} \sum_{j=1}^{J} \sum_{m=1}^{J} \nu \sigma_{jm} (a_{ij} - a_{im})^2$$

where σ_{jm} is a measure of purely spatial proximity between the SAR j is adjacent to the SAR m (the other parameters are described in the paper). We defined $\sigma_{jm} = 1$ when the SARs j and m are spatially adjacent, and $\sigma_{jm} = 0$ otherwise.

First, we implemented a cross-validation approach as in the previous sections to select the best value of ν, i.e. the value that maximizes the criteria defined in the section 4.1.1:

```
## The neighbouring matrix
pg <- neig2mat(nb2neig(poly2nb(SAR)))

## We calculate the effort again
vv <- sapply(1:length(SAR@data$codeSAR), function(i) {
    mean(Vj1[as.character(dfdata$SAR) == as.character(SAR@data$codeSAR)[i]])
})
vv2 <- sapply(1:length(SAR@data$codeSAR), function(i) {
    mean(dfdata$Sj[as.character(dfdata$SAR) == as.character(SAR@data$codeSAR)[i]])
})
dfVj <- data.frame(nom = factor(SAR@data$codeSAR),
    Vj = vv, Sj = vv2)


## Then we make sure that the variable SAR in the
## data.frame scsldetect has the exact same set of
## levels as the first column of dfVj
scsldetect$SAR <- factor(scsldetect$SAR, levels = levels(dfVj[,
    1]))
## And we make sure that the other vectors are
## factors
scsldetect$Species <- factor(scsldetect$Species)
scsldetect$Status <- factor(scsldetect$Status)
scsldetect$Year <- factor(scsldetect$Year)

cvpraspa <- crossvalidation(scsldetect$Species, scsldetect$SAR,
    scsldetect$Status, scsldetect$Year, dfVj, vecnu = seq(0,
        0.6, by = 0.1), proximities = pg, control = list(stopCrit = 1e-06))
```

We now examinate the global results:

```
glospa <- global.crossvalCisstat(cvpraspa)
plot(glospa)
```

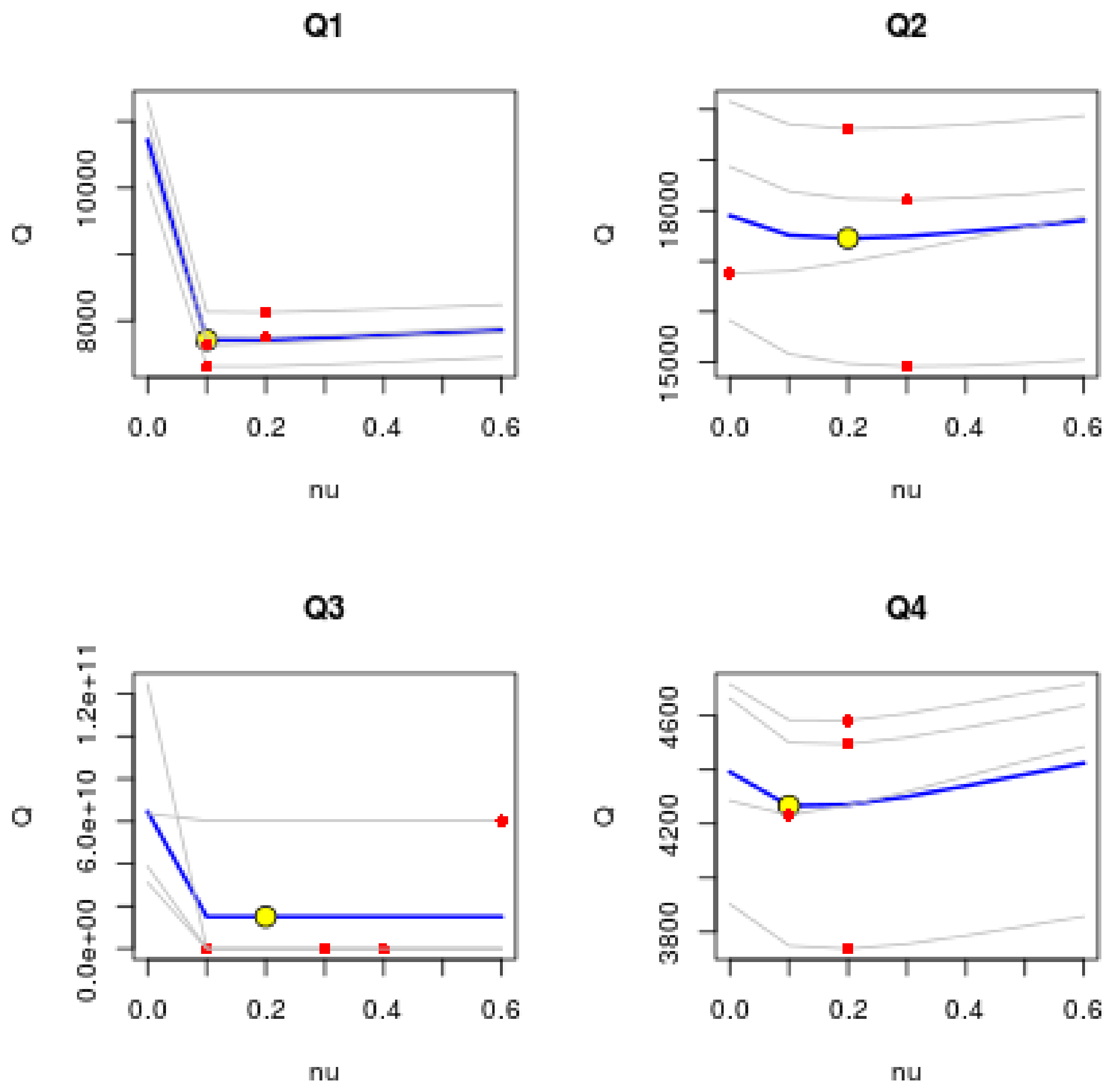

In this case, the value $\nu = 0.2$ was characterized by a small prediction error. We considered this value to fit our purely spatial model.

7.2 Comparison of a purely spatial regularization with the spatial+environmental regularization

To identify the most suitable regularization type (purely spatial or spatial+environmental), we have implemented a cross-validation approach. We have considered the even years (2002, 2004) and the odd years (2003, 2005) separately. We have fitted a model using the data collected during the even years and we have measured the prediction error using our four criteria on the odd years, and conversely. We thus used an approach identical to the approach implemented in section 6.1. We first prepared the data for this comparison:

```
## We first prepare the data
scsldetect$Year <- as.numeric(as.character(scsldetect$Year))
scsl <- scsldetect
effortpercar <- effortpercar
linumsar <- linumsar
cars <- carsscsl
```

```
#### 1. Sampling effort of the dead supposed to be
#### constant within a department

## model 1
g <- 1
obsa <- scsl[scsl$Year%%2 == (g - 1), ]
obsb <- scsl[scsl$Year%%2 != (g - 1), ]
veha <- cars[cars$Year%%2 == (g - 1), ]
vehb <- cars[cars$Year%%2 != (g - 1), ]

## calculation of the effort for odd and even years
## (Vja and Vjb)
Vja <- sapply(as.character(SAR@data$codeSAR), function(y) {
    ## the department code
    x <- substr(y, 1, 2)
    ## remember that the departments 91, 95 and 78 are
    ## coded 78:
    if (any(c("91", "95") == x))
        x <- "78"
    ## Calculation of the total effort in the
    ## department, standardized by the area
    a <- veha$Car[as.character(veha$Department) ==
        x]
    b <- veha$Km[as.character(veha$Department) == x]
    if (all(is.na(b))) {
        b <- carsscsl$Km[as.character(carsscsl$Department) ==
            x]
    }
    c <- department@data$Area[department@data$Code ==
        x]
    length(a) * mean(na.omit(b))/c
})
Vjb <- sapply(as.character(SAR@data$codeSAR), function(y) {
    ## the department code
    x <- substr(y, 1, 2)
    ## remember that the departments 91, 95 and 78 are
    ## coded 78:
    if (any(c("91", "95") == x))
        x <- "78"
    ## Calculation of the total effort in the
    ## department, standardized by the area
    a <- vehb$Car[as.character(vehb$Department) ==
        x]
    b <- vehb$Km[as.character(vehb$Department) == x]
    if (all(is.na(b))) {
        b <- carsscsl$Km[as.character(carsscsl$Department) ==
            x]
    }
    c <- department@data$Area[department@data$Code ==
        x]
    length(a) * mean(na.omit(b))/c

})
Vjab <- sapply(as.character(dfdata$SAR), function(x) Vja[names(Vja) ==
    x])
Vjbb <- sapply(as.character(dfdata$SAR), function(x) Vjb[names(Vjb) ==
```

```
    x])

## construction of the calibration and validation
## datasets
dfdatasara <- do.call("rbind", lapply(levels(factor(scsl$Species)),
    function(i) {
        ## For each species, we keep only the detected
        ## animals of the species
        obse <- obsa[obsa$Species == i, ]

        ## For the dead and living animals
        do.call("rbind", lapply(c("D", "L"), function(k) {
            ## and for each sar
            do.call("rbind", lapply(SAR@data$codeSAR,
                function(sara) {
                  ## return the number of detected animals belonging
                  ## to dataset a
                  data.frame(Nijk = nrow(obse[as.character(obse$SAR) ==
                    sara & obse$Status == k, ]), Sp = i,
                    Sta = k, SAR = sara)
                }))
        }))
    }))

dfdatasarb <- do.call("rbind", lapply(levels(factor(scsl$Species)),
    function(i) {
        ## For each species, we keep only the detected
        ## animals of the species
        obse <- obsb[obsb$Species == i, ]

        ## For the dead and living animals
        do.call("rbind", lapply(c("D", "L"), function(k) {
            ## and for each sar
            do.call("rbind", lapply(SAR@data$codeSAR,
                function(sara) {
                  ## return the number of detected animals belonging
                  ## to dataset b
                  data.frame(Nijk = nrow(obse[as.character(obse$SAR) ==
                    sara & obse$Status == k, ]), Sp = i,
                    Sta = k, SAR = sara)
                }))
        }))
    }))


surf <- SAR@data$Area
surfb <- sapply(as.character(dfdata$SAR), function(x) {
    surf[as.character(SAR@data$codeSAR) == x]
})
dfdatasara <- data.frame(dfdatasara, Vj = Vjab, Area = surfb)
dfdatasarb <- data.frame(dfdatasarb, Vj = Vjbb, Area = surfb)
dfdatasara$Sp <- factor(dfdatasara$Sp)
dfdatasarb$Sp <- factor(dfdatasarb$Sp)
dfdatasara$SAR <- factor(dfdatasara$SAR)
dfdatasarb$SAR <- factor(dfdatasarb$SAR)
dfdatasara$Sta <- factor(dfdatasara$Sta)
dfdatasarb$Sta <- factor(dfdatasarb$Sta)
```

We then fitted the models for odd and even years respectively, conditional on the matrix of spatial proximities (using the effort measure $V_j^{(1)}$)

```
## Model fit
modpraa <- penalizedmodel(dfdatasara$Sp, dfdatasara$SAR,
    dfdatasara$Sta, dfdatasara$Nijk, dfdatasara$Vj,
    Area = dfdatasara$Area, proximities = pg, nu = 0.2,
    control = list(typealgo = 3, verbose = TRUE, stopCrit = 1e-07))
modprab <- penalizedmodel(dfdatasarb$Sp, dfdatasarb$SAR,
    dfdatasarb$Sta, dfdatasarb$Nijk, dfdatasarb$Vj,
    Area = dfdatasarb$Area, proximities = pg, nu = 0.2,
    control = list(typealgo = 3, verbose = TRUE, stopCrit = 1e-07))
```

```
load("palc/modpraaspa.Rdata")
load("palc/modprabspa.Rdata")
```

We then calculated the different criteria measuring the prediction error for this purely spatial model:

```
## Prediction for the dead
preb <- dfdatasarb[dfdatasarb$Sta == "D", ]
prea <- dfdatasarb[dfdatasara$Sta == "D", ]
Yhatbfroma <- sapply(1:length(preb$Sp), function(i) {
    exp(modpraa$aij[preb$SAR[i], preb$Sp[i]] + log(preb$Vj[i]))
}) * preb$Area
Yhatafromb <- sapply(1:length(prea$Sp), function(i) {
    exp(modprab$aij[prea$SAR[i], prea$Sp[i]] + log(prea$Vj[i]))
}) * prea$Area


## Prediction error according to the four criteria
Q1 <- sum(-dpois(preb$Nijk, Yhatbfroma, log = TRUE))
Q2 <- sum((Yhatbfroma - preb$Nijk)^2)
Q3 <- sum(((Yhatbfroma - preb$Nijk)^2)/Yhatbfroma)
Q4 <- sum(((Yhatbfroma - preb$Nijk)^2)/(Yhatbfroma +
    1))
critbfroma <- c(Q1, Q2, Q3, Q4)

Q1 <- sum(-dpois(prea$Nijk, Yhatafromb, log = TRUE))
Q2 <- sum((Yhatafromb - prea$Nijk)^2)
Q3 <- sum(((Yhatafromb - prea$Nijk)^2)/Yhatafromb)
Q4 <- sum(((Yhatafromb - prea$Nijk)^2)/(Yhatafromb +
    1))
critafromb <- c(Q1, Q2, Q3, Q4)
aaspa <- rbind(critafromb, critbfroma)
aaspa <- as.data.frame(rbind(aaspa, apply(aaspa, 2,
    mean)))
names(aaspa) <- c("Q1", "Q2", "Q3", "Q4")
row.names(aaspa) <- c("EvenFromOdd", "OddFromEven",
    "Mean")
res1 <- (prea$Nijk - Yhatafromb)/sqrt(Yhatafromb)
res2 <- (preb$Nijk - Yhatbfroma)/sqrt(Yhatbfroma)
```

We now present the results of this cross-validation approach for the spatial+environmental regularization (already displayed in section 6.2) and for the purely spatial approach respectively:

```
## Spatial+environmental
aa

##                Q1    Q2   Q3   Q4
## EvenFromOdd 4388  5979 2588 1104
## OddFromEven 6092 31266 8063 4060
## Mean        5240 18623 5326 2582

## Purely spatial
aaspa

##                Q1    Q2   Q3   Q4
## EvenFromOdd 4460  6424 2723 1187
## OddFromEven 6098 31133 7978 4058
## Mean        5279 18779 5350 2622
```

Thus, it is clear that the spatial and environmental regularization used in our paper gives smaller prediction errors than the purely spatial approach. This demonstrates that it is important to account for the environmental similarity between SARs when estimating the relative densities of the species of interest.

We therefore considered, in the rest of this document, the model fitted in the section 4.2 (i.e. the model fitted using both spatial and environmental regularization, using the effort measure $V_j^{(1)}$). We study this model in detail in the next section.

8 Model examination

8.1 Model residuals and overdispersion

In this section, we studied the residuals of the model:

```
res <- residuals(modVj1)
pre <- predict(modVj1)
## We present only the predicted values >0
## (residuals associated to predicted 0 are all = 0)
plot(pre[pre > -20], res[pre > -20], xlab = "predicted log-densities",
    ylab = "standardized residuals")
```

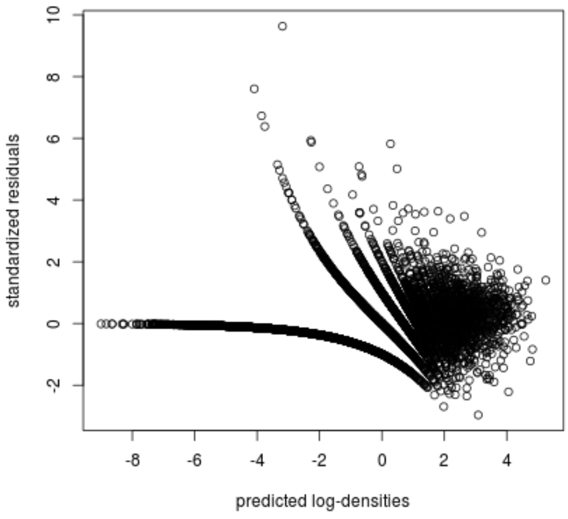

These residuals do not present any problematic pattern, except a very small number standardized residuals greater than 6. This indicates a very minor overdispersion in the data. We studied more precisely this overdispersion in our data.

Thus, a first check of the model consists in plotting the absolute value of the Pearson's residuals as a function of the predicted values. Indeed, the fact that a variable Y follows a Poisson distribution implies that:

$$\text{Var}(Y) = E(Y)$$

We have calculated the standardized Pearson's residuals (see p. 141 in CAMERON et TRIVEDI, 1998), which are expected to have a constant variance when plotted as a function of the predicted values. Therefore, the absolute value of the standardized residuals are expected to have a constant mean, when plotted as a function of the predicted values. We show this plot below, which allows to identify possible relationships of the form:

$$\text{Var}(Y) = E(Y)^h$$

with $h > 1$. We also added a lowess smoothing to this plot, to enhance the visualization of the relationship between these two quantities:

```
plot(exp(pre[pre > -20]), abs(res[pre > -20]), xlab = "predicted densities",
    ylab = "|standardized residuals|", xlim = c(0,
        110))
lines(lowess(abs(res[pre > -20]) ~ exp(pre[pre > -20])),
    col = "red", lwd = 2)
```

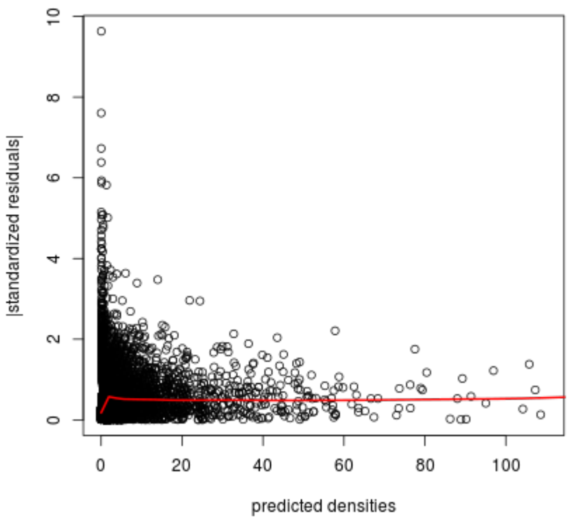

The standardized residuals clearly have a constant variance! However, the constant variance of the residuals on this plot could have been obtained if:

$$\text{Var}(Y) = h \times E(Y)$$

with $h > 1$. We therefore calculated the dispersion parameter h. Thus, we calculated the Pearson's statistic (sum of squares of Pearson's residuals), along with the number of degrees of freedom:

```
chi2pearson <- sum(res^2)
numberDegreesFreedom <- length(res) - length(c(modVj1$aij)) -
    length(modVj1$ejk[, 1]) - length(modVj1$pi2)
resultsover <- round(c(Chi2Pearson = chi2pearson, NDegreesFreedom = numberDegreesFreedom))
resultsover

##     Chi2Pearson NDegreesFreedom
##            5962            4913
```

This confirms the presence of a minor amount of over dispersion. The dispersion parameter h for this model can therefore be estimated therefore estimated with χ^2/d (McCullagh et Nelder, 1989, p. 175), i.e.

```
resultsover[1]/resultsover[2]

## Chi2Pearson
##       1.214
```

Thus, if we considered the overdispersion in our model, the standard errors of the estimated parameters would be inflated by $100 \times (\sqrt{1.2} - 1) \approx 10\%$, which is negligible given the other sources of error in our model (Note that Lindsey (1999) proposes, as a rule of thumb, that the overdispersion should be accounted for when the estimated dispersion parameter is lower than 2: in our case, the dispersion parameter is much lower than 2!). Moreover, note that the bootstrap approach that we used to estimate the precision of our estimates (see section 8.3) would allow to account for the overdispersion in the estimation of the precision if it was present in the data.

It is clear that the Poisson model is a reasonable model for the process studied here.

Moreover, the residuals did not show any problematic structure when plotted according to the species, the status or the SAR:

```r
par(mfrow = c(3, 1))
boxplot(res ~ dfdata$Sp, xlab = "Species", ylab = "standardized residuals")
boxplot(res ~ dfdata$Sta, xlab = "Status", ylab = "standardized residuals")
boxplot(res ~ dfdata$SAR, xlab = "Status", ylab = "standardized residuals")
```

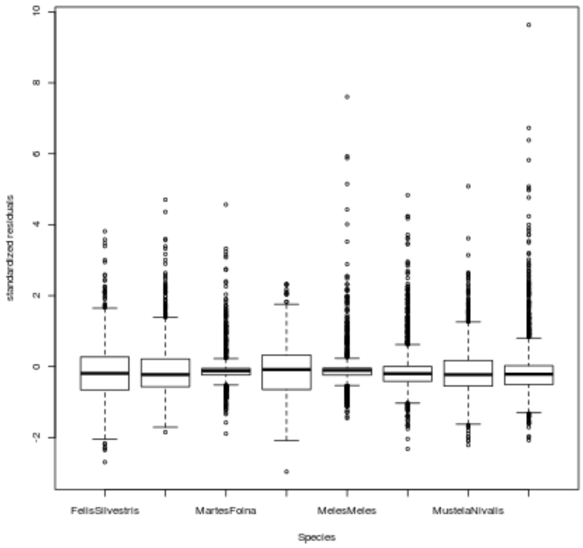
standardized residuals
Species
FelisSilvestris
MartesFoina
MelesMeles
MustelaNivalis

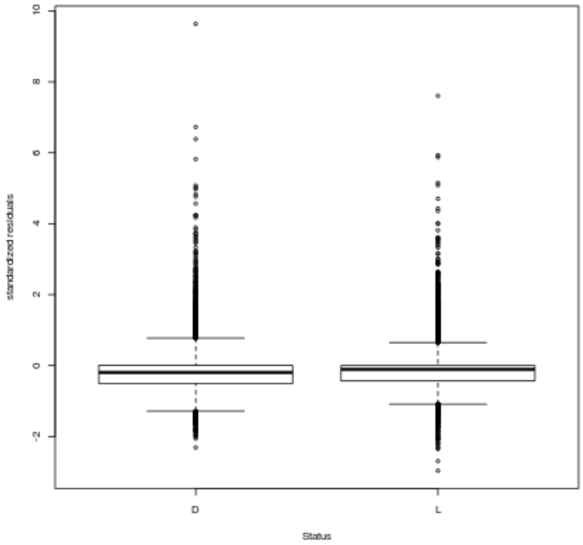
standardized residuals
Status
D
L

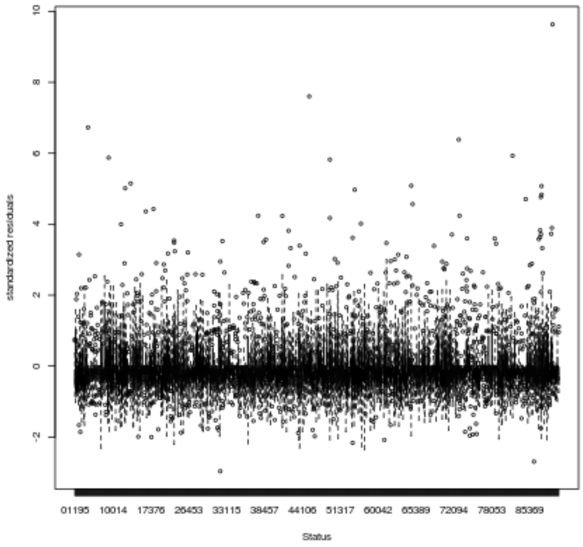
standardized residuals
Status
01195 10014 17376 26453 33115 38457 44106 51317 60042 65389 72094 78053 85369

8.2 Goodness of fit

We present in this section a plot showing for each species and each status, the relation between the predicted number of detections according to the model and the actual number of detections:

```
pre <- exp(predict(modVj1))
X <- attr(modVj1, "X")
Y <- attr(modVj1, "Y")
par(mfrow = c(2, 8))
tmp <- lapply(c("D", "L"), function(sta) {
    lapply(unique(scsldetect$Species), function(sp) {
        plot(Y[X$Sp == sp & X$Sta == sta], pre[X$Sp ==
            sp & X$Sta == sta], xlab = "Observation",
            ylab = "Prediction", main = paste(sp, sta))
        abline(0, 1, lwd = 2, col = "red")
    })
})
```

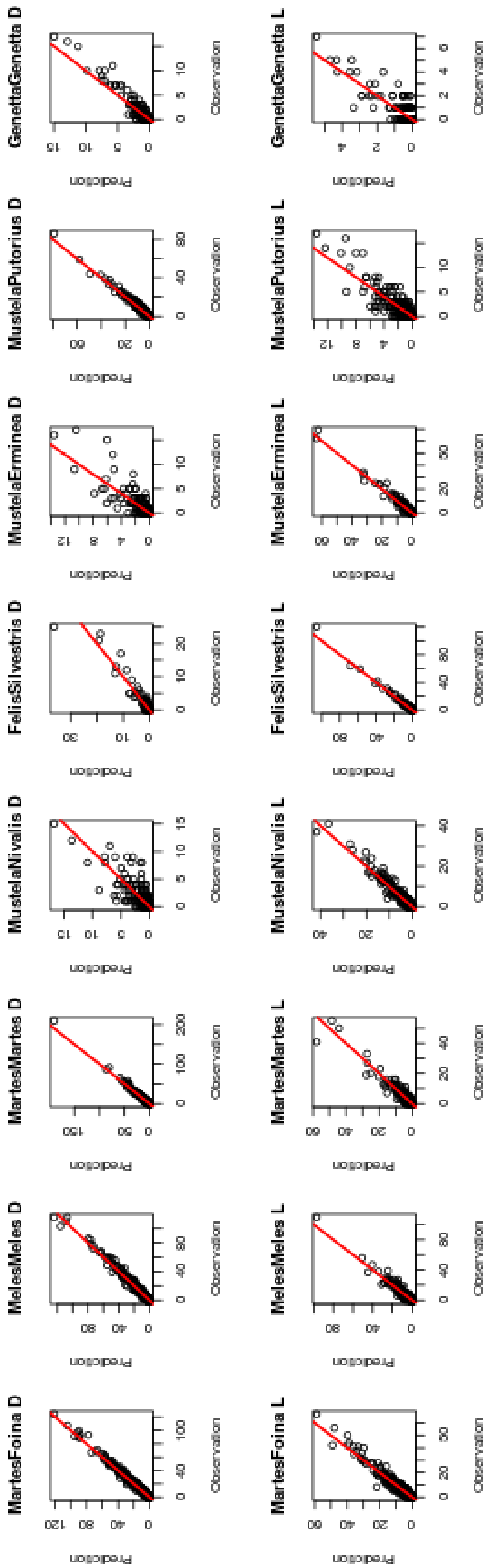
GenettaGenetta D
MustelaPutorius D
MustelaErminea D
FelisSilvestris D
MustelaNivalis D
MartesMartes D
MelesMeles D
MartesFoina D
GenettaGenetta L
MustelaPutorius L
MustelaErminea L
FelisSilvestris L
MustelaNivalis L
MartesMartes L
MelesMeles L
MartesFoina L
Prediction
Observation

The red line corresponds to the line $y = x$. We can see that the model fit is correct for all species and all status.

8.3 Coefficients of variation associated to the estimates

Finally, we have estimated the standard errors associated with the estimates with a bootstrap approach. We used the function `bootstrapcisstat` (see the help page of this function) to estimate these standard errors (100 bootstrap samples have been drawn to calculate these standard errors):

```
vv <- sapply(1:nrow(SAR), function(i) {
    mean(Vj1[as.character(dfdata$SAR) == as.character(SAR@data$codeSAR)[i]])
})
vv2 <- sapply(1:nrow(SAR), function(i) {
    mean(dfdata$Sj[as.character(dfdata$SAR) == as.character(SAR@data$codeSAR)[i]])
})
dfVj <- data.frame(nom = factor(as.character(SAR@data$codeSAR)),
    Vj = vv, Sj = vv2)
scsldetect$SAR <- factor(scsldetect$SAR, levels = levels(dfVj[,
    1]))

boopra <- bootstrapcisstat(factor(scsldetect$Species),
    scsldetect$SAR, factor(scsldetect$Status), dfVj,
    nu = 0.3, proximities = prx, B = 100, control = list(typealgo = 4,
        stopCrit = 1e-07, StartingValues = list(aij = modVj1$aij,
            ej2 = modVj1$ejk[, 2], pi2 = modVj1$pi2)))
```

The coefficient of variation of the density estimates are mapped below:

```
## calculates the SE
ssddpra <- sdaij(boopra, type = "response")

## calculates the coefficient of variation
cvm <- lapply(1:ncol(ssddpra), function(i) (100 * ssddpra[,
    i]/exp(modVj1$aij[, i])))

## plots the coefficient of variation
par(mfrow = c(3, 3), mar = c(0, 0, 2, 0))
tmp <- sapply(1:8, function(i) {
    plot(SAR, col = grey((8 - as.numeric(cut(cvm[[i]],
        breaks = c(0, 10, 20, 30, 40, 50, 60, 500))))/8),
        border = NA)
    title(levels(factor(scsldetect$Species))[i])
})

## The scale
plot.new()
plot.window(xlim = c(0, 1), ylim = c(0, 8), xaxs = "i",
    yaxs = "i")
limitsxy <- 0:7
limits <- c(0, 10, 20, 30, 40, 50, 60, 500)
colors <- grey((8 - as.numeric(cut((limits[-length(limits)] +
    limits[-1])/2, breaks = c(0, 10, 20, 30, 40, 50,
    60, 500))))/8)
rect(0.3, limitsxy[-length(limitsxy)], 0.5, limitsxy[-1],
    col = colors)
aa <- paste(limits[-length(limits)], "% - ", limits[-1],
    "%", sep = "")
```

```
aa[length(aa)] <- ">60%"
text(0.55, (limitsxy[-length(limitsxy)] + limitsxy[-1])/2,
    aa, pos = 4)
```

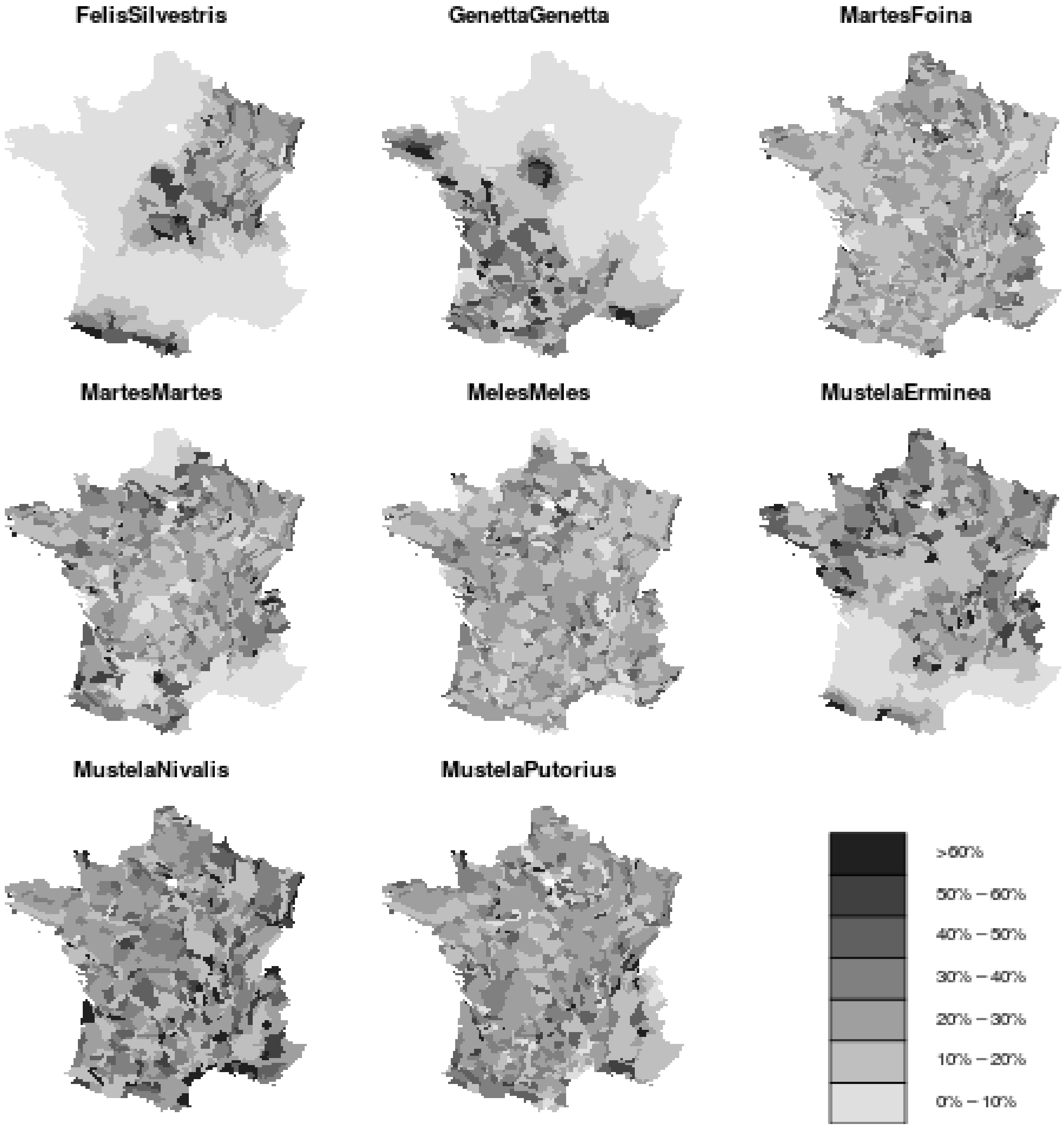

We calculated the interquartile interval of the distribution of coefficient of variation for the species of interest:

```
iq <- sapply(cvm, quantile, c(0.25, 0.75))
colnames(iq) <- names(modVj1$pi2)
round(iq[, 3:8])

##     MartesFoina MartesMartes MelesMeles
## 25%          15           13         15
## 75%          30           33         32
```

```
##     MustelaErminea MustelaNivalis MustelaPutorius
## 25%             13             19              16
## 75%             33             38              35
```

This gives a general idea of the precision obtained with our approach. In general, the coefficient of variation of the relative density estimate in a SAR is comprised approximately between 15% and 35%. Therefore, we should not expect to draw conclusions on fine changes in density with this approach.

9 Addendum: purely spatial model

As we noted in the main text, it is possible to use our modelling framework to smooth the spatial distribution patterns of the species of interest. Thus, we replaced the spatial and environmental proximity metrics used in our regularization by a purely spatial proximity, with $\pi_{jm} = 1$ when SARs j and m are adjacent, and $\pi_{jm} = 0$ otherwise. We calculated this proximity matrix below:

```
pg <- neig2mat(nb2neig(poly2nb(SAR)))
```

Then, we fitted our model again, setting the parameter ν to a very large value ($\nu = 20$) to study the large scale distribution patterns of the species over the study area:

```
spamod <- penalizedmodel(factor(dfdata$Sp), factor(dfdata$SAR),
    factor(dfdata$Sta), dfdata$Nijk, Vj1, Area = dfdata$Sj,
    proximities = pg, nu = 20, control = list(typealgo = 4,
        verbose = TRUE, stopCrit = 1e-07))
```

These highly smoothed maps are displayed below:

```
par(mfrow = c(3, 3), mar = c(0, 0, 2, 0))
tmp <- sapply(1:8, function(i) {
    plot(SAR, col = grey(togrey(-as.numeric(cut2(spamod$aij[,
        i], g = 20)))), border = NA)
    title(levels(factor(dfdata$Sp))[i])
})
```

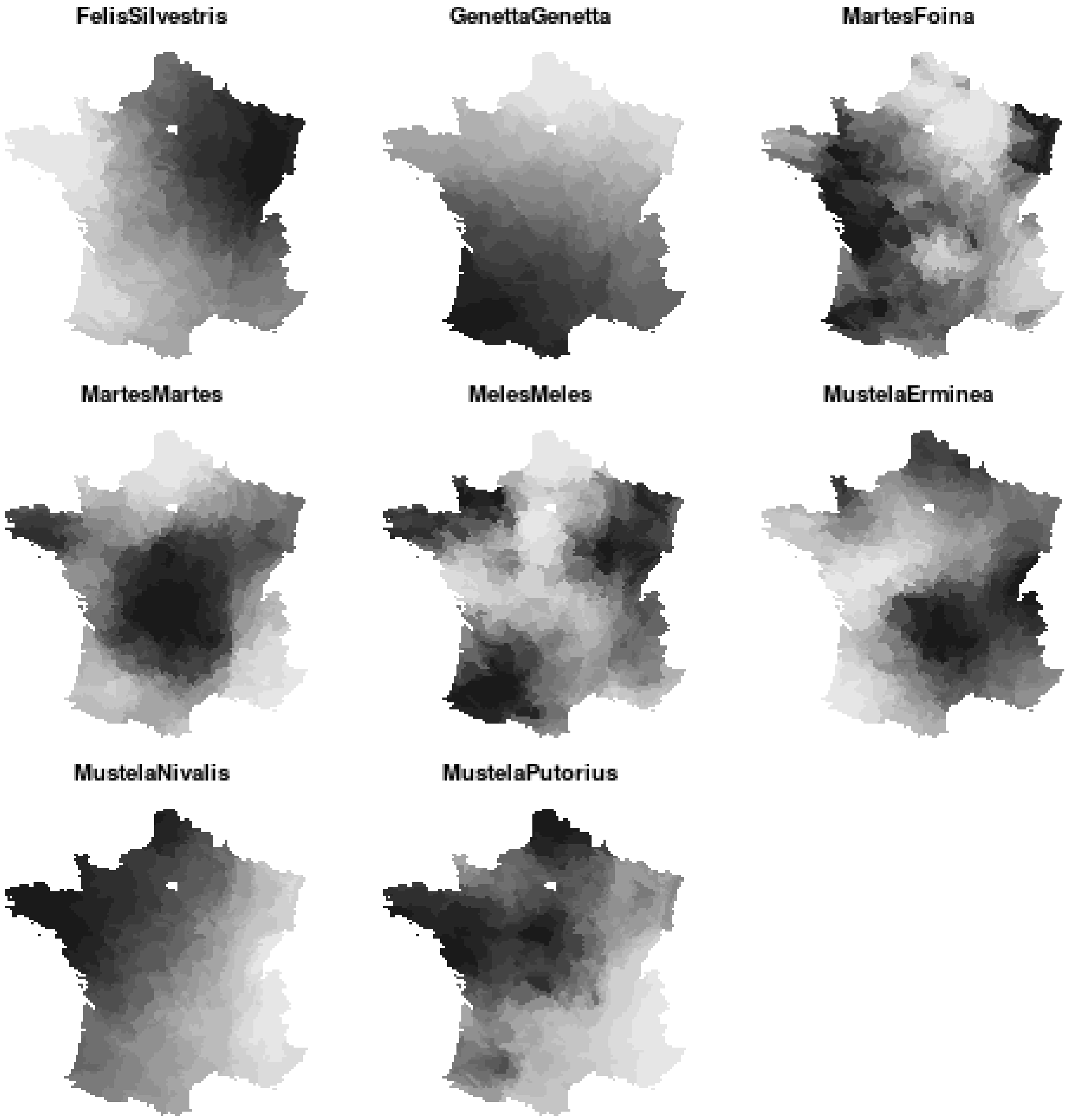

References

CAMERON, A. et TRIVEDI, P. (1998). *Regression analysis of count data.* Econometric society Monographs, No 30.

LAMPORT, L. (1994). *LaTeX. A document preparation system. User's guide and Reference manual.* Addison-Wesley Publishing compagny.

LINDSEY, J. (1999). On the use of corrections for overdispersion. *Journal of the Royal Statistical Society: Series C (Applied Statistics)*, 48(4): 553–561.

MCCULLAGH, P. et NELDER, J. (1989). *Generalized linear models. Second Edition.* Chapman & Hall, London.

NOCEDAL, J. et WRIGHT, S. (2006). *Numerical Optimization. Second edition.* Springer.

XIE, Y. (2013). knitr: A general-purpose package for dynamic report generation in R. *R package version 1.2*.
